# Characterization of Intracellular Precore-Derived Proteins and Their Functions in Hepatitis B Virus-Infected Human Hepatocytes

**DOI:** 10.1128/mbio.03501-22

**Published:** 2023-01-30

**Authors:** Xupeng Hong, Emily N. Bianchini, Joseph Che-Yen Wang, Jianming Hu

**Affiliations:** a Department of Microbiology and Immunology, The Pennsylvania State University College of Medicine, Hershey, Pennsylvania, USA; The University of North Carolina at Chapel Hill

**Keywords:** hepatitis B virus, precore, core, replication, infection, HBV, chimpanzee, primary human hepatocytes

## Abstract

Hepatitis B virus (HBV) precore protein is not essential for viral replication but is thought to facilitate chronic infection. In addition to the secreted precore products, including the hepatitis B e antigen (HBeAg) and PreC protein, intracellular precore-derived proteins in HBV-infected human hepatocytes remain poorly characterized, and their roles, if any, remain largely unknown. Here, we detected multiple precore derivatives, including the nonprocessed precursor p25 and the processing intermediate p22, in HBV-infected human hepatocytes as well as human hepatoma cells overexpressing the HBV precore protein. Both p25 and p22 showed phosphorylated and unphosphorylated forms, which were located in different intracellular compartments. Interestingly, precore expression was associated with decreases in intracellular HBV core protein (HBc) and secreted DNA-containing virions but was also associated with an increase in secreted empty virions. The decrease in HBc by precore could be attributed to cytosolic p22, which caused HBc degradation, at least in part by the proteasome, and consequently decreased HBV pregenomic RNA packaging and DNA synthesis. In addition, cytosolic p22 formed chimeric capsids with HBc in the cell, which were further secreted in virions. In contrast, the PreC antigen, like HBeAg, was secreted via the endoplasmic reticulum (ER)-Golgi secretory pathway and was thus unable to form capsids in the cell or be secreted in virions. Furthermore, p25, as well as p22, were secreted in virions from HBV-infected human hepatocytes and were detected in the sera of HBV-infected chimpanzees. In summary, we have detected multiple intracellular precore-derived proteins in HBV-infected human hepatocytes and revealed novel precore functions in the viral life cycle.

## INTRODUCTION

**FIG 1:**
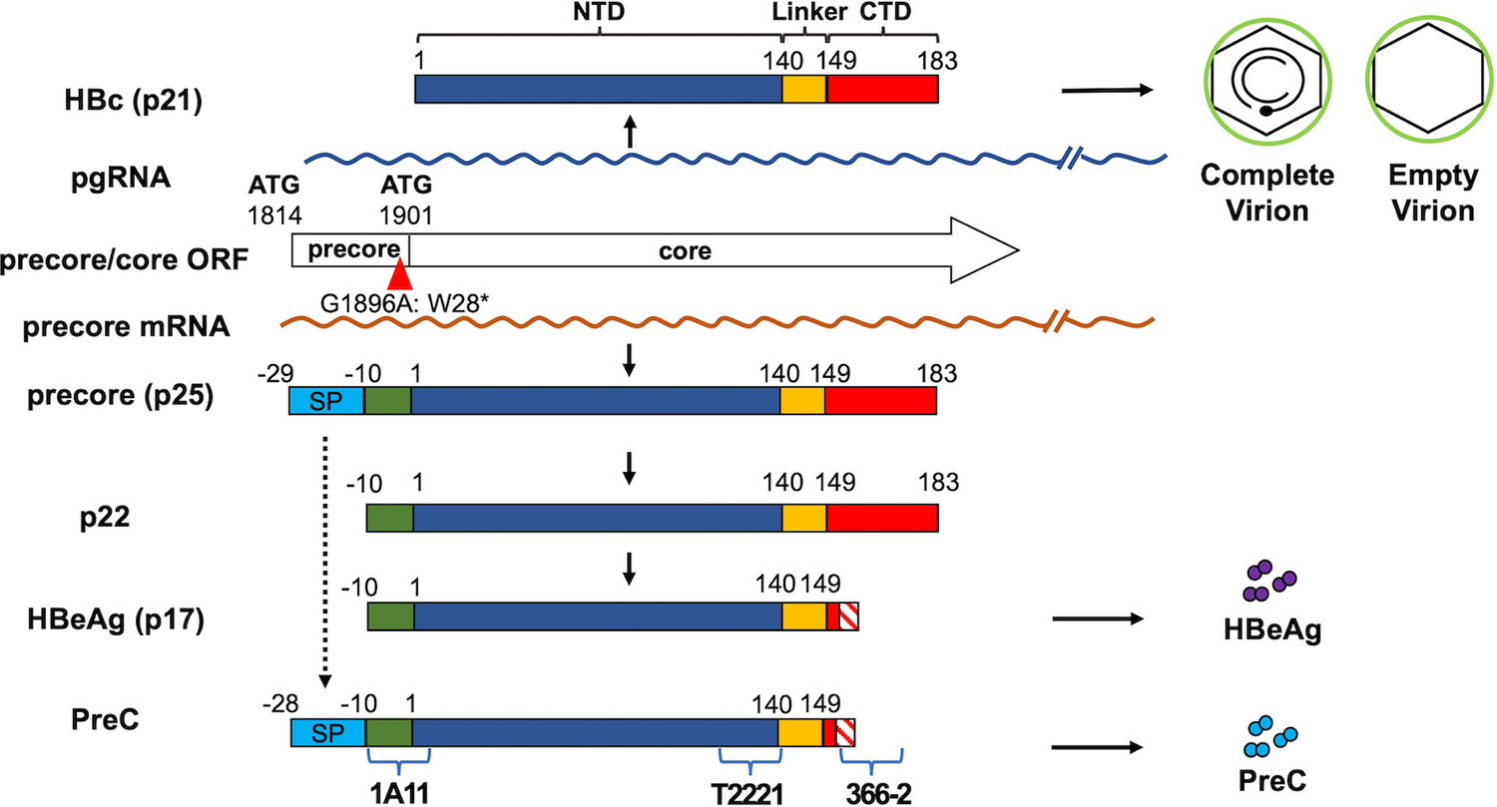
Biosynthesis of HBc and HBV precore protein products. HBV precore and core proteins are expressed from the overlapping precore/core ORFs but using two distinct mRNA templates. HBc, translated from pgRNA, forms the icosahedral capsid inside complete and empty virions. The precore mRNA, which has a 29-aa extension at the 5′ end relative to pgRNA and contains the first initiation codon in the precore/core genes, is the template for translation of the precore protein (p25), the precursor to HBeAg and PreC. The N-terminal signal peptide (SP) of p25 is removed by the signal peptidase cotranslationally during the translocation of p25 into the ER lumen, leading to the production of p22, which is further processed at its CTD before being secreted as the dimeric HBeAg (p17). PreC, which has the noncleaved SP but processed CTD, is also secreted. The exact C-terminal processing sites of HBeAg and PreC are heterogeneous, as indicated by the hashed boxes. The epitopes recognized by the monoclonal antibody (MAb) 1A11, specific to precore-derived proteins (epitope: −10 to 5 aa), MAb T2221 (epitope: 130 to 140 aa), specific to the NTD shared by all precore/core gene products, and MAb 366-2 (epitope: 150 to 164 aa), specific to the CTD of HBc and some precore proteins (p22 and p25), are indicated (blue brackets).

An estimated 295 million people worldwide are chronically infected with the hepatitis B virus (HBV) (1), a small, enveloped DNA virus belonging to the family *Hepadnaviridae*. HBV exploits the human sodium taurocholate cotransporting polypeptide (huNTCP) as its entry receptor (2). After entering hepatocytes, the HBV relaxed circular DNA (rcDNA) is converted into the covalently closed circular DNA (cccDNA), the template for making viral genomic (i.e., pregenomic RNA [pgRNA]) and subgenomic RNAs, inside the nucleus of infected hepatocytes (3). The HBV genome contains four overlapping open reading frames (ORFs), namely, precore/core, polymerase (RT), surface, and X (4). The 3.5-kb pgRNA is the template for reverse transcription in the production of progeny rcDNA and also the template for translation of the RT and core protein (HBc) (Fig. 1). The RT and pgRNA are packaged together into a capsid formed by HBc, wherein pgRNA is reverse transcribed to single-stranded DNA (ssDNA) and then rcDNA by RT (4). Nucleocapsids containing rcDNA are either secreted as complete virions after envelopment with viral surface proteins (HBsAg) or recycled back to the nucleus to replenish the cccDNA pool (5). In addition, empty capsids (i.e., without viral genomes) are also formed in the cell by HBc alone and after envelopment are secreted as empty virions, which are typically 100-fold more abundant than complete virions in the blood of HBV-infected patients or in the supernatant of HBV-replicating cell cultures (6, 7).

The precore/core gene has two in-frame start codons and encodes HBc and the precore protein, respectively, using two distinct RNA templates (Fig. 1). HBc (p21), translated from pgRNA, is the structural subunit of viral capsids. In contrast, the precore protein translated from the precore mRNA, which is extended slightly at the 5′ end relative to pgRNA so as to contain the first initiation codon in the precore/core gene, is not essential for viral replication and is thought to facilitate viral persistence (8–12). Interestingly, the precore protein is conserved in all orthohepadnaviruses infecting mammals and avihepadnaviruses infecting birds (13–15), suggesting a critical role of precore protein in hepadnaviral infection.

The precore protein precursor is a 25-kDa protein (p25), which has a 29-amino acid (aa) precore-specific extension at the N-terminal domain (NTD) compared with HBc (Fig. 1). A 19-aa hydrophobic signal peptide at the NTD precore-specific region directs p25 translocation into the endoplasmic reticulum (ER) lumen cotranslationally. A 22-kDa precore protein processing intermediate (p22) is then generated from p25 after signal peptide removal (16, 17) (Fig. 1). Subsequently, the C-terminal domain (CTD) of p22 is further processed by a furin-like endoprotease in the *trans*-Golgi network to produce a 17-kDa hepatitis B e antigen (HBeAg) that is secreted as a homodimer (18, 19) (Fig. 1). In addition to HBeAg, another secreted precore product, PreC (previously known as p22cr), with noncleaved signal peptide but a processed CTD, has been found in the blood of HBV-infected patients and the culture supernatant of HBV-infected or precore-expressing cells (20–22). Although PreC was initially reported to be secreted in empty virions (20), our recent studies have shown that it is not part of the virion and instead has a density and size similar to HBeAg, that is, likely also secreted as a dimer (21). However, the biogenesis pathway of PreC remains unclear. Besides these two secreted precore gene products (i.e., HBeAg and PreC), p22 and p25 have been detected intracellularly in cells either overexpressing the precore gene alone or transfected with replication-competent HBV DNAs (replicons) (23–26) as well as in the liver of precore transgenic mice (27). In contrast to p22 in the ER lumen (i.e., the precursor of HBeAg), which is unphosphorylated (28), the cytosolic p22, which is generated either due to abortive ER translocation or retrotranslocation back to the cytosol following signal peptide cleavage from p25, is phosphorylated (16, 28–30). In addition, a phosphorylated p25 (P-p25), the precore protein precursor that likely is never translocated into the ER lumen, was reported in precore-expressing cells (24). Importantly, it remains unclear if any of these intracellular precore-derived proteins are present in HBV-infected human hepatocytes, the natural host cells for HBV.

The biological functions of intracellular precore proteins remain poorly understood, including the potential roles of these precore protein derivatives in the HBV life cycle. Overexpression of the precore protein has been shown to suppress HBV replication in the transgenic mouse model (27). Interestingly, the HBV G1896A variant, which converts the 28th codon of the precore protein from tryptophan to a stop codon and thus abolishes precore protein expression and hence HBeAg secretion (Fig. 1) (31), is thought to be associated with fulminant hepatitis (32). The G1896A mutation was reported to increase pgRNA encapsidation and DNA replication compared with wild-type (WT) HBV in replicon-transfected hepatoma cells (33, 34). Furthermore, cytosolic p22 was shown to negatively regulate HBV replication, possibly by forming chimeric capsids with HBc (33). However, the presence of the putative chimeric capsids was based solely on cosedimentation of p22 and HBc following sucrose gradient ultracentrifugation (33). Another study shows that HBc was coimmunoprecipitated with precore, suggesting a specific interaction between HBc and precore protein (35), but it was unclear whether HBc and p22 formed true chimeric capsids or some other forms of complexes. Importantly, the functions of the precore proteins in HBV-infected human hepatocytes have yet to be elucidated.

In this study, we have characterized intracellular precore-derived proteins and investigated their functions in the HBV life cycle by using HBV-infected primary human hepatocytes (PHHs; the most physiologically relevant model for studying HBV infection *in vitro*), HBV-infected human hepatoma cells, and human hepatoma cells overexpressing the precore proteins. We detected, for the first time, multiple intracellular precore protein species in HBV-infected PHHs and demonstrated that intracellular precore protein derivatives decreased intracellular HBc levels and differentially modulated the secretion of complete versus empty virions. We verified the presence of chimeric capsids containing HBc and p22 and, furthermore, detected such chimeric capsids in secreted virions. These results have thus demonstrated the expression of the precore proteins in HBV-infected human hepatocytes, the natural host cells of HBV, and revealed novel functions of the precore proteins in the HBV life cycle.

## RESULTS

### Multiple intracellular precore-derived proteins are detected in HBV-infected PHHs and precore-transfected human hepatoma cells.

**FIG 2:**
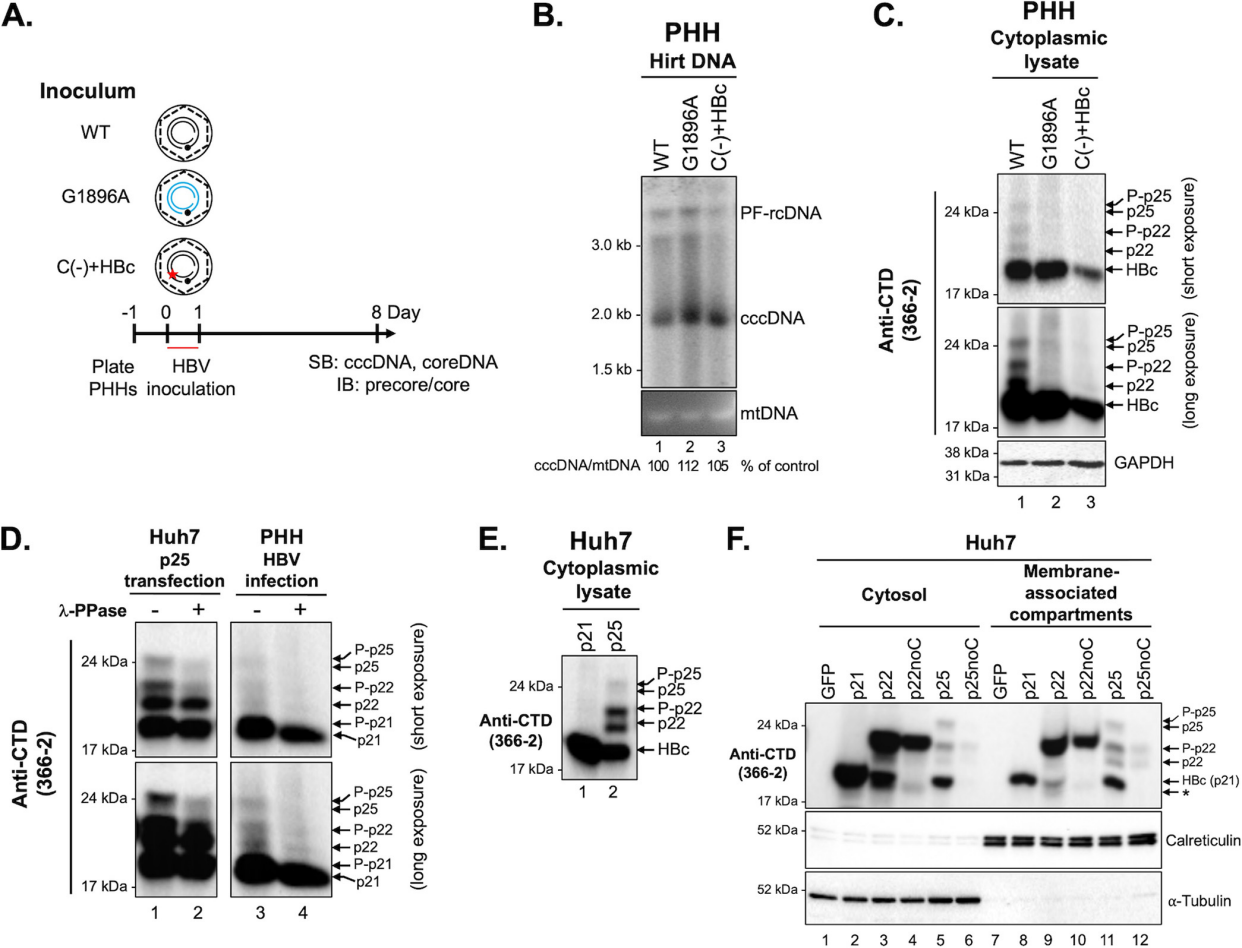
Multiple intracellular precore-derived proteins were detected in HBV-infected PHHs and precore-transfected human hepatoma cells. (A) The scheme of the HBV infection experiment in PHHs. PHHs were plated 1 day before infection and infected at an MOI of 400 genome equivalents per cell (GE/cell) with WT, G1896A, and C(-)+HBc virus prepared from transfected HepG2 cells. WT and G1896A viruses were prepared from the replicon-transfected HepG2 cells, and C(-)+HBc virus was prepared from HepG2 cells cotransfected with pCIΔA-HBV-HBc-C(-) and pCI-HBc. Eight days postinfection (dpi), the infected cells were harvested for analysis of HBV cccDNA, core DNA, and precore/core protein expression. (B) HBV protein-free DNA (PF-DNA) was extracted from infected PHHs by the Hirt extraction method and analyzed by Southern blotting using a ^32^P-labeled HBV DNA probe. Mitochondrial DNA (mtDNA), visualized by ethidium bromide staining, was used as the loading control, and the levels of cccDNA are indicated at the bottom as a percentage of WT. (C) The cytoplasmic lysates of HBV-infected PHHs were analyzed by SDS-PAGE followed by Western blotting using anti-HBV precore/core CTD MAb 366-2. A short exposure (top) and a long exposure (middle) are presented. Western blot analysis of GAPDH was used as the loading control (bottom). (D) Cytoplasmic lysates of precore (p25)-transfected Huh7 cells (harvested at 72 h posttransfection) and HBV-infected PHHs (harvested at 8 dpi) were treated with λ-phosphatase at 30°C overnight and analyzed by Western blotting using MAb 366-2. (E) Huh7 cells were transfected with HBc (p21) and precore (p25) and harvested at 72 h posttransfection. Cytoplasmic lysates were analyzed by Western blotting using MAb 366-2. (F) Huh7 cells were transfected with HBc (p21), the precore intermediate p22 (or p22noC), or the precore precursor p25 (or p25noC) expression constructs. In contrast to p25 (or p25noC), which is the authentic precore expression construct, expression of p22 occurs in the cytosol due to the lack of signal peptide on p22 (or p22noC). To avoid the internal initiation of HBc (p21) expression from p22 and p25 (21), we mutated the internal HBc initiation codon ATG (Met) to GTG (Val) in the p22 and p25 expression constructs, which are named p22noC and p25noC. Seventy-two hours posttransfection, the cells were harvested and subjected to subcellular fractionation. Cytosolic and membrane-associated precore/core proteins were detected by Western blotting using MAb 366-2. The cytosolic and membrane fractions of p21, p22, and p22noC were loaded 4-fold less than those of p25 and p25noC, and the corresponding fractions from green fluorescent protein (GFP; i.e., precore/core-negative)-transfected cells were used for adjustment of the loading volume. Calreticulin and α-tubulin served as loading controls for the membrane and cytosolic fractions, respectively.

To characterize precore protein expression in HBV-infected PHHs, we analyzed PHH cytoplasmic lysates at 8 days postinfection (dpi) (Fig. 2A). We included the HBV G1896A mutant as the negative control for the detection of precore-derived proteins and HBV C(-)+HBc as the negative control for *de novo* HBc synthesis. HBV C(-)+HBc harbors a genome defective in *de novo* HBc expression and hence cannot support replication but remains competent for precore protein synthesis and secretion. All these viruses showed similar infectivity, as evidenced by similar levels of cccDNA at 8 dpi (Fig. 2B). We detected four precore-derived proteins in WT HBV-infected PHHs, which were absent in the G1896A mutant-infected cells (Fig. 2C). Interestingly, these intracellular precore-derived proteins were only detected at lower levels in HBV C(-)+HBc-infected cells than in WT HBV-infected cells (Fig. 2C; Fig. S1A in the supplemental material; see also Fig. 2F and Fig. S1C), whereas the extracellular precore-derived proteins, HBeAg and PreC, were at similar levels (Fig. S1A). According to their mobilities, we tentatively assigned these precore-derived proteins as the phosphorylated (P-p25 and P-p22) and unphosphorylated p25 and p22, respectively. Furthermore, we found that the brefeldin A (BFA) treatment, which blocks ER to Golgi translocation, significantly decreased HBeAg and PreC levels in the cell culture supernatant (Fig. S1A), consistent with the requirement of the ER-Golgi secretory pathway for the secretion of PreC as well as HBeAg. In contrast, intracellular levels of the putative unphosphorylated p22 and p25, but not P-p22 and P-p25, were increased after BFA treatment (Fig. S1A), suggesting that unphosphorylated p22 and p25 were likely the precursors of HBeAg and PreC, respectively. As these proteins would be in the secretory pathway, they would not be phosphorylated due to a lack of protein kinase activity there. Our assignment of these precore species was consistent with a recent study showing that unphosphorylated p22 in the membrane, but not cytosolic P-p22, is the precursor of HBeAg (28). In addition, we found that naked capsid release, as detected by the native agarose gel electrophoresis (NAGE) assay, was not affected by BFA treatment (Fig. S1B). Because of the low levels of precore-derived proteins in HBV-infected PHHs, λ-phosphatase treatment only showed a decrease in P-p25 and P-p22 but no significant increase in unphosphorylated p25 and p22, which was more clearly shown in p25-transfected Huh7 cells (Fig. 2D; see later also). In addition, we were able to better resolve the putative phosphorylated and unphosphorylated p25 in infected PHHs by using high-resolution sodium dodecyl sulfate-polyacrylamide gel electrophoresis (SDS-PAGE) (Fig. S1A).

10.1128/mbio.03501-22.1 FIG S1 Effects of brefeldin A treatment on HBV precore/core protein secretion. (A) PHHs were plated 1 day before infection and infected at an MOI of 400 genome equivalents per cell (GE/cell) with WT, G1896A, and C(-)+HBc virus prepared from transfected HepG2 cells. Seven dpi, PHHs were treated without (lanes 1 to 4) or with (lanes 5 to 8) 1 μg/mL brefeldin A (BFA) for 24 h. Cell culture supernatant was then collected at 8 dpi, concentrated (25×), and resolved by SDS-PAGE followed by immunoblotting using anti-precore/core NTD MAb T2221 and anti-PreS2 MAb S26. At the same time, the cytoplasmic lysates were resolved by regular (a gel height of 12.5 cm) and high-resolution (a gel height of 35 cm) SDS-PAGE, followed by immunoblotting using anti-precore/core CTD MAb 366-2. A short exposure and a long exposure of high-resolution SDS-PAGE membranes are presented. (B) Concentrated culture supernatants from HBV-infected PHHs described in A were resolved by native agarose gel electrophoresis (NAGE) and transferred to nitrocellulose membranes. HBV DNA, capsid in virions, and HBsAg associated with virions and subviral particles were detected subsequentially using a ^32^P-labeled HBV DNA probe, anti-HBV precore/core NTD MAb T2221, anti-HBV precore/core CTD MAb 366-2, and polyclonal anti-HBs antibody on the same membrane. (C) Huh7 cells were transfected with pCI-HBc (p21), pCI-p25 (p25), and pCI-p25-noC (p25-noC). At 48 h posttransfection, the cells were treated without (lanes 1 to 3) or with (lanes 4 to 6) 0.25 μg/mL BFA for 24 h. Cell culture supernatant was then collected, concentrated (25×), and resolved by SDS-PAGE, followed by immunoblotting using anti-precore/core NTD MAb T2221 and anti-CTD MAb 366-2. The cytoplasmic lysates were resolved by regular SDS-PAGE, followed by immunoblotting using anti-precore/core CTD MAb 366-2. V, virion; HBe, HBV e antigen; PreC, HBV PreC antigen; HBs, HBV surface antigen. Download FIG S1, JPG file, 0.9 MB. Copyright © 2023 Hong et al. 2023 Hong et al. https://creativecommons.org/licenses/by/4.0/ This content is distributed under the terms of the Creative Commons Attribution 4.0 International license.

To help characterize the intracellular precore protein species further, we also expressed precore alone in transfected Huh7 cells and were able to detect the four intracellular precore-derived proteins more clearly than in HBV-infected PHHs due to higher expression levels in the transfected cells (Fig. 2E). We again confirmed these precore species by BFA (Fig. S1C) and λ-phosphatase treatment (Fig. 2D). In addition, we found that unphosphorylated p22 and p25 were primarily in membrane-associated compartments (Fig. 2F), further confirming that p22 and p25 were the precursors of HBeAg and PreC, respectively, and were in the secretory pathway. Interestingly, both P-p22 and P-p25 were found in both the cytosolic and membrane-associated compartments, as was HBc (p21) (Fig. 2F). It is likely that some P-p22 and P-p25, phosphorylated in the cytosol, were associated with autophagosomes, as was recently reported (36). Altogether, we detected phosphorylated and unphosphorylated p22 and p25 in both HBV-infected PHHs and precore-transfected hepatoma cells.

As reported by us and others (21, 33), appreciable amounts of HBc (p21) could be expressed from either the p25 or p22 expression construct (Fig. 2E and F; Fig. S1C) due to the presence of the HBc initiation codon following the precore specific sequences (Fig. 1). To eliminate HBc expression from the precore expression constructs so that only precore-derived proteins were expressed, we removed the HBc initiation codon from the p25 and p22 expression constructs to make construct p25noC and p22noC, respectively (see Materials and Methods for details). The lack of HBc expression from p25noC and p22noC was confirmed by SDS-PAGE and Western blotting (Fig. 2F; Fig. S1C). Furthermore, as observed earlier in infected PHHs (Fig. 2C; Fig. S1A), the lack of HBc led to decreased levels of precore-derived proteins expressed from p25noC and p22noC compared to p25 and p22 (Fig. 2F; Fig. S1C).

### Loss of precore is associated with increased levels of intracellular HBc and secreted DNA-containing virions but decreased levels of empty virions.

**FIG 3:**
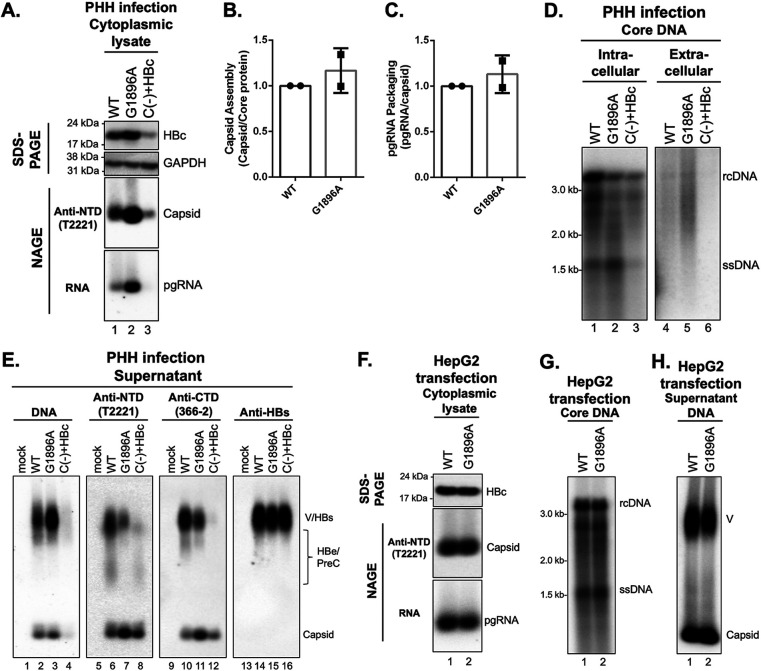
Effects of the precore protein on HBV infection in PHHs and HBV replication in human hepatoma cells. (A) Levels of HBc proteins (top) were measured by Western blotting using the MAb 366-2 after SDS-PAGE of cytoplasmic lysates from the infected PHHs. GAPDH was used as the loading control. The assembled capsids (middle) and packaged pgRNA (bottom) were detected by the MAb T2221 and the antisense HBV RNA probe, respectively, following the resolution of cytoplasmic lysates from the infected PHHs by native agarose gel electrophoresis (NAGE) and transfer to a nitrocellulose membrane. (B) Capsid assembly efficiency was determined by normalizing the levels of capsids to those of total HBc protein, with the efficiency of WT set to 1.0. (C) pgRNA packaging efficiency was determined by normalizing the levels of pgRNA to those of capsids, with the efficiency of WT set to 1.0. (D) HBV core DNA was released from the cytoplasmic lysate or concentrated supernatant of HBV-infected PHHs (harvested at 8 dpi) by SDS-proteinase K treatment and detected by Southern blotting. The supernatant was concentrated by 25× using a 10-kDa cutoff ultrafiltration unit. (E) The concentrated PHH culture supernatant was resolved by NAGE and transferred to a nitrocellulose membrane. HBV DNA, capsid (naked or in virions), and envelope proteins in virions and subviral particles were detected sequentially using a ^32^P-labeled HBV DNA probe, anti-HBV precore/core NTD MAb T2221, anti-HBV precore/core CTD MAb 366-2, and anti-HBs polyclonal antibody, respectively, on the same membrane. (F to H) The HBV replicon construct expressing the WT or G1896A mutation was transfected into HepG2 cells. Levels of HBc proteins (top) were measured by SDS-PAGE and Western blotting using MAb T2221 (F). The assembled capsids (middle) and packaged pgRNA (bottom) were detected by the anti-precore/core NTD MAb T2221 and anti-sense HBV RNA probe, respectively, following the resolution of cytoplasmic lysates by NAGE and transfer to a nitrocellulose membrane. HBV core DNA was released from the nucleocapsids inside the cytoplasm of transfected HepG2 cells by SDS-proteinase K treatment and detected by Southern blotting (G). Culture supernatant from HBV replicon-transfected HepG2 cells was harvested at day 5 posttransfection, concentrated (50×), resolved by NAGE, and transferred to a nitrocellulose membrane (H). HBV DNA was detected with a ^32^P-labeled DNA probe. pgRNA, pregenomic RNA; HBc, HBV core protein; rcDNA, relaxed circular DNA; ssDNA, single-stranded DNA; V, virion; HBe, HBV e antigen; PreC, HBV PreC antigen; HBs, HBV surface antigen.

To study the effects of precore protein on HBV replication, we first analyzed HBc expression in the cytoplasmic lysates of infected PHHs. We found that G1896A mutant-infected cells showed increased HBc levels compared with WT HBV-infected cells (Fig. 3A, top). Interestingly, HBc was also detectable from cells infected with the HBV C(-)+HBc inoculum (Fig. 3A, top), indicating that some of the input HBc from the incoming virions (or naked capsids that are routinely released from HBV DNA-transfected hepatoma cells used to make the inoculum) remained associated with the cells (either on the cell surface or inside the cells) for at least 8 days. In addition, we found that G1896A-infected cells showed increased levels of capsid and encapsidated pgRNA compared with WT HBV-infected cells (Fig. 3A, middle and bottom). However, after normalization of capsid levels to HBc protein levels as an indicator for capsid assembly efficiency and normalization of pgRNA packaging levels to capsid levels as an indicator of pgRNA packaging efficiency, both WT and G1896A-mutant viruses showed similar efficiency in capsid assembly and pgRNA packaging (Fig. 3B and C). Therefore, the increased capsid and encapsidated pgRNA levels in G1896A mutant-infected cells were primarily due to increased HBc expression. Our results thus indicated that a major effect of the precore protein in the infected PHHs was to decrease intracellular HBc levels.

We then measured levels of nucleocapsid-associated HBV DNA (i.e., core DNA) from cytoplasmic lysates of infected PHHs. We found that G1896A-mutant virus showed increased immature DNA species (i.e., ssDNA) compared with WT virus (Fig. 3D), which could be accounted for by the increased encapsidated pgRNA levels in G1896A-infected cells (Fig. 3A). In contrast, WT HBV-infected cells showed higher levels of more mature DNA species, including the rcDNA and the partially double-stranded DNA (dsDNA) species that migrated between rcDNA and ssDNA, than G1896A mutant-infected cells (Fig. 3D). Our previous study suggested that these partially dsDNA species, like rcDNA, is likely derived from the envelope-protected nucleocapsids (i.e., intracellular virions) yet to be secreted extracellularly (37). Furthermore, we found more mature DNA species in the supernatant of G1896A-infected cells than in the supernatant of WT HBV-infected PHHs (Fig. 3D), suggesting that G1896A enhanced/accelerated the secretion of DNA-containing virions. Consistently, G1896A HBV-infected PHHs showed increased levels of DNA-containing virions compared to WT HBV-infected PHHs, as revealed by the NAGE assay (Fig. 3E). HBV C(-)+HBc-infected PHHs failed to support *de novo* HBc synthesis but still showed some weak signals of DNA-containing naked capsids, likely derived from the inoculum input. In contrast, G1896A-infected PHHs showed decreased levels of virion-associated core protein signal, representing primarily empty virions (6), despite high levels of intracellular capsids, suggesting that the loss of precore expression led to decreased secretion of empty virions but enhanced secretion of complete virions. The increased levels of naked capsids released by G1896A-infected PHHs compared with WT HBV-infected PHHs could be accounted for by increased intracellular capsids (Fig. 3E). In addition, we found similar results in HBV-infected HepG2-NTCP cells (Fig. S2A to E), except that the G1896A mutation enhanced secretion of both complete and empty virions (see Discussion below).

10.1128/mbio.03501-22.2 FIG S2 Multiple intracellular precore-derived proteins were detected in HBV-infected and precore-transfected human hepatoma cells. (A) The scheme of an HBV infection assay in HepG2-huNTCP cells. HepG2-huNTCP cells were plated 3 days before infection and infected at a MOI of 400 genome equivalents per cell (GE/cell) with WT, G1896A, and C(-)+HBc virus prepared from transfected HepG2 cells. Eight dpi, the infected cells were harvested for analysis of HBV cccDNA, core DNA, and precore/core protein expression. (B) HBV protein-free DNA (PF-DNA) was extracted from infected HepG2-huNTCP cells by the Hirt extraction method and analyzed by Southern blotting without (lanes 1 to 3) or with pretreatment by exonuclease (Exo) I and III (lanes 4 to 6). Mitochondrial DNA (mtDNA) was used as the loading control, which was eliminated by Exo I/III treatment as expected (lanes 4 to 6, bottom). The levels of cccDNA are indicated at the bottom as a percentage of WT. (C) Levels of HBc proteins (top) were measured by Western blotting using the MAb 366-2 after SDS-PAGE of cytoplasmic lysates from the infected HepG2-huNTCP cells. GAPDH was used as the loading control. The assembled capsids (middle) and packaged pgRNA (bottom) were detected by the MAb T2221 and the anti-sense HBV RNA probe, respectively. (D) HBV core DNA was released from the cytoplasmic lysate or concentrated supernatant (by 50×) from HBV-infected HepG2-huNTCP cells collected at 8 dpi by SDS-proteinase K treatment and detected by Southern blotting. (E) Concentrated HepG2-huNTCP culture supernatant was resolved by NAGE and transferred to a nitrocellulose membrane. HBV DNA and capsid in virions were detected subsequentially using a ^32^P-labeled HBV DNA probe, anti-HBV precore/core NTD MAb T2221, and anti-HBV precore/core CTD MAb 366-2, respectively, on the same membrane. (F to H) The HBV replicon construct expressing the WT or G1896A mutation was transfected into Huh7 cells. Levels of HBc proteins (top) were measure by SDS-PAGE and Western blotting using MAb T2221 (F). The assembled capsids (middle) and packaged pgRNA (bottom) were detected by the anti-precore/core NTD MAb T2221 and anti-sense HBV RNA probe, respectively, following the resolution of cytoplasmic lysates by NAGE and transferred to a nitrocellulose membrane. HBV core DNA was released from the nucleocapsids inside the cytoplasm by SDS-proteinase K treatment and detected by Southern blotting (G). Culture supernatant from HBV replicon-transfected Huh7 cells was harvested at day 5 posttransfection, concentrated (50×), resolved by NAGE, and transferred to a nitrocellulose membrane (H). HBV DNA was detected by a ^32^P-labeled DNA probe. pgRNA, pregenomic RNA; HBc, HBV core protein; rcDNA, relaxed circular DNA; ssDNA, single-stranded DNA; V, virion; HBe, HBV e antigen; PreC, HBV PreC antigen. Download FIG S2, JPG file, 0.7 MB. Copyright © 2023 Hong et al. 2023 Hong et al. https://creativecommons.org/licenses/by/4.0/ This content is distributed under the terms of the Creative Commons Attribution 4.0 International license.

Our results thus far indicate that the G1896A mutant shows increased levels of HBc expression and enhanced secretion of complete virions but reduced secretion of empty virions. To test whether these phenotypes were due to potential effects of the G1896A mutation in *cis* (i.e., acting on pgRNA or DNA) or to the lack of precore protein expression, we introduced the same mutation in the context of the HBV DNA replicon that supports pgRNA expression after transfection into hepatoma cells but not precore protein expression. After replicon transfection, we found that the G1896A mutant showed no effects on levels of HBc protein, capsid, pgRNA packaging, DNA synthesis, or DNA-containing virion secretion compared with the WT (Fig. 3F to H; Fig. S2F to H). These transfection results thus excluded any *cis* effects of the G1896A mutation. Together, the results above demonstrate that the precore protein reduces HBc expression and decreases complete virion secretion in infected cells.

### Intracellular precore proteins decrease HBc expression.

**FIG 4:**
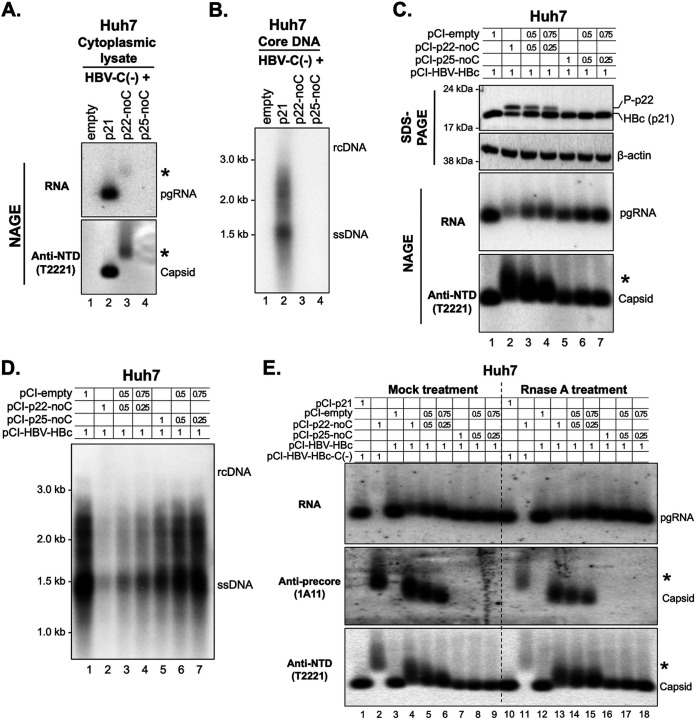
Effects of p22 and p25 on HBV replication in human hepatoma Huh7 cells. (A) Huh7 cells cultured in 35-mm dishes were cotransfected with pCI-HBV-HBc-C(-) (i.e., HBV-C[-]) and pCI-HBc (p21), pCI-p22-noC, or pCI-p25-noC, as indicated, at a 1:1 mass ratio and harvested at 72 h posttransfection. Cytoplasmic lysate was resolved by NAGE and transferred to a nitrocellulose membrane. HBV RNA associated with capsids (top) was detected by anti-sense HBV RNA probe, followed by immunoblotting using anti-precore/core NTD MAb T2221 (bottom). (B) HBV core DNA released from the cytoplasmic nucleocapsids that were described in A was treated with SDS-proteinase K and detected by Southern blotting. (C) Huh7 cells cultured in 35-mm dishes were cotransfected with HBV replicon construct pCI-HBV-HBc and pCI-empty, pCI-p22-noC, or pCI-p25-noC construct at the indicated amounts (in μg). The cells were harvested at 72 h posttransfection. Intracellular HBV precore and core proteins were detected by Western blotting using anti-HBV precore/core NTD MAb T2221, and β-actin was used as the loading control (top). The assembled capsids and packaged pgRNA (bottom) were detected by the anti-precore/core NTD MAb T2221 and anti-sense HBV RNA probe, respectively, following the resolution of cytoplasmic lysates by NAGE and transfer to a nitrocellulose membrane. (D) HBV core DNA released from the cytoplasmic nucleocapsids that were described in C was treated with SDS-proteinase K and detected by Southern blotting. (E) Cytoplasmic lysates described in A and C were treated with or without RNase A (final concentration: 200 μg/mL) for 30 min at 37°C. The samples were resolved by NAGE and transferred to a nitrocellulose membrane, followed by the detection of HBV RNA using the anti-sense HBV RNA probe and Western blotting of precore protein using anti-precore MAb 1A11 and of precore/core protein using MAb T2221. pgRNA, pregenomic RNA; HBc, HBV core protein; rcDNA, relaxed circular DNA; ssDNA, single-stranded DNA; *, complexes of p22-RNA.

To test whether intracellular precore proteins cause decreased HBc levels, we cotransfected the plasmid construct expressing p22, the cytosolic form of the precore intermediate, or p25, the authentic precore precursor protein, along with the HBV replicon into the human hepatoma cell lines Huh7 and HepG2. Consistent with previous findings (21, 25), neither p22 nor p25 supported detectable HBV replication (Fig. 4A and B; Fig. S3A and B). We detected weak HBV RNA and strong precore/core protein signals that migrated slower than the capsid (denoted by an asterisk) representing putative p22-RNA complexes (see later). Intriguingly, we found that increased p22 levels were associated with decreased HBc levels when we cotransfected p22 or p25 and the WT HBV replicon at various ratios in Huh7 and HepG2 cells (Fig. 4C; Fig. S3C). Even with cytosolic p22 (i.e., P-p22) that was undetectable by SDS-PAGE and Western blotting, when p25 and the HBV replicon were cotransfected, HBc levels were still decreased (Fig. 4C; Fig. S3C), suggesting that even very low levels of cytosolic p22 could effectively decrease HBc levels, potentially by inducing HBc degradation.

10.1128/mbio.03501-22.3 FIG S3 Effects of precore protein on HBV replication in human hepatoma HepG2 cells. (A) HepG2 cells were cotransfected with pCI-HBV-HBc-C(-) (i.e., HBV-C[-]) and pCI-HBc (p21), pCI-p22-noC, or pCI-p25-noC, as indicated, at a 1:1 mass ratio and harvested at 72 h posttransfection. Cytoplasmic lysate was resolved by NAGE and transferred to a nitrocellulose membrane. HBV RNA encapsidated in capsids (top) was detected by antisense HBV RNA probe, followed by immunoblotting using anti-precore/core NTD MAb T2221 (bottom). (B) HBV core DNA released from the cytoplasmic nucleocapsids from the samples that were described in A was treated with SDS-proteinase K and detected by Southern blotting. (C) HepG2 cells were cotransfected with HBV replicon construct pCI-HBV-HBc and pCI-empty, pCI-p22-noC, or pCI-p25-noC construct at the indicated amounts. The cells were harvested at 72 h posttransfection. Intracellular HBV precore and core proteins were detected by Western blotting using anti-HBV precore/core NTD MAb T2221, and β-actin was used as the loading control (top). The assembled capsids and packaged pgRNA (bottom) were detected by the anti-precore/core NTD MAb T2221 and anti-sense HBV RNA probe, respectively, following the resolution of cytoplasmic lysates by NAGE and transfer to a nitrocellulose membrane. (D) HBV core DNA released from the cytoplasmic nucleocapsids from the samples described in C was treated with SDS-proteinase K and detected by Southern blotting. (E) Cytoplasmic lysates described in A and C were treated with or without RNase A (a final concentration of 200 μg/mL) for 30 min at 37°C. The samples were resolved by NAGE and transferred to a nitrocellulose membrane followed by the detection of HBV RNA using the antisense HBV RNA probe and Western blotting of precore protein using anti-precore MAb 1A11 and of precore/core protein using MAb T2221. pgRNA, pregenomic RNA; HBc, HBV core protein; rcDNA, relaxed circular DNA; ssDNA, single-stranded DNA. Asterisks (*) indicate complexes of p22-RNA. Download FIG S3, JPG file, 0.7 MB. Copyright © 2023 Hong et al. 2023 Hong et al. https://creativecommons.org/licenses/by/4.0/ This content is distributed under the terms of the Creative Commons Attribution 4.0 International license.

To explore the possible mechanisms of p22-induced HBc reduction, we treated cells with a proteasomal inhibitor (MG132) or lysosomal inhibitor (chloroquine). We found that inhibition of the proteasome, but not lysosome, partially restored HBc levels in Huh7 cells in the presence of p22 (Fig. S4A), suggesting that p22 might be degraded together with HBc (e.g., as a p22-p21 complex) at least partially via proteasomes. However, p22 was equally distributed between the cytoplasm and nucleus, while HBc was more abundant in the cytoplasm than in the nucleus (Fig. S4B). Furthermore, when coexpressing p22 and HBc, p22 enhanced nuclear localization of HBc (Fig. S4C), likely due to the inhibition of capsid assembly as a result of the formation of p22-HBc heterocomplexes (see later). In addition, we found that the HBc protein and HBV RNA signals from cells cotransfected with p22 and the HBV replicon showed reduced mobilities on the NAGE assay compared with those from cells without p22 or p25 expression (Fig. 4C and Fig. S3C, lower panels, lanes 2 to 4 and 5 to 7 versus lane 1), suggesting that HBc and p22 might form slow-migrating chimeric capsids and at least some of the chimeric capsids could support pgRNA packaging. Moreover, the capsid-associated pgRNA signal was positively correlated with HBc protein levels but was negatively correlated with p22 levels (Fig. 4C and Fig. S3C lanes 2 to 7). As p22 by itself could not support detectable capsid assembly or pgRNA packaging (Fig. 4A; Fig. S3A), these results suggested that the reduced pgRNA packaging was due to decreased HBc levels. Furthermore, we found that core DNA levels were generally correlated with pgRNA packaging levels (Fig. 4D; Fig. S3D), suggesting that the reduction of HBV DNA replication by p22 was due to reduction of pgRNA packaging, which was in turn due to decreased HBc expression as well as the inhibition of capsid assembly by p22 (see later). Similarly, we observed the same effects of p22 or p25 expression in HepG2 cells (Fig. S3), but the variations in the mobility of capsids and capsid-associated RNA were more significant in HepG2 cells than in Huh7 cells.

10.1128/mbio.03501-22.4 FIG S4 Effects of cytosolic p22 on HBc expression. (A) Huh7 cells were cotransfected with pCI-HBc (p21), pCI-p22-noC (p22noC), or pCI-empty (pCI), as indicated, at a 1:1 mass ratio. At 48 h posttransfection, cells were treated with 50 μM chloroquine or 1 μM MG132 for 24 h, as indicated. Cytoplasmic lysates were then collected and resolved by regular SDS-PAGE and detected with anti-precore/core NTD MAb T2221 (top). β-Actin was used as the loading control (middle), and the lipidation of LC3B (LC3B-II) was used to indicate successful treatment with chloroquine (58). (B) Huh7 cells were transfected with pCI-HBc (p21) and pCI-p22 (p22). Cells were harvested at 72 h posttransfection and subjected to subcellular fractionation using a subcellular protein fractionation kit (Thermo Fisher). The cytosolic and nuclear fractions were resolved by SDS-PAGE followed by immunoblotting using anti-precore/core NTD MAb T2221. β-Actin and lamin A/C were used as loading controls for the cytosolic and nuclear fractions, respectively. (C) Huh7 cells were cotransfected with pCI-HBV-HBc (HBV), pCI-p22-noC (p22noC), or pCI-empty (pCI), as indicated, at a 1:1 mass ratio. Cells were harvested at 72 h posttransfection. The whole-cell lysates (W), cytoplasmic lysates (C), and nuclear lysates (N) were resolved by regular SDS-PAGE and detected with anti-precore/core NTD MAb T2221 (top). α-Tubulin and lamin A/C were used as loading controls for the cytoplasmic and nuclear fractions, respectively. Download FIG S4, JPG file, 0.4 MB. Copyright © 2023 Hong et al. 2023 Hong et al. https://creativecommons.org/licenses/by/4.0/ This content is distributed under the terms of the Creative Commons Attribution 4.0 International license.

**FIG 5:**
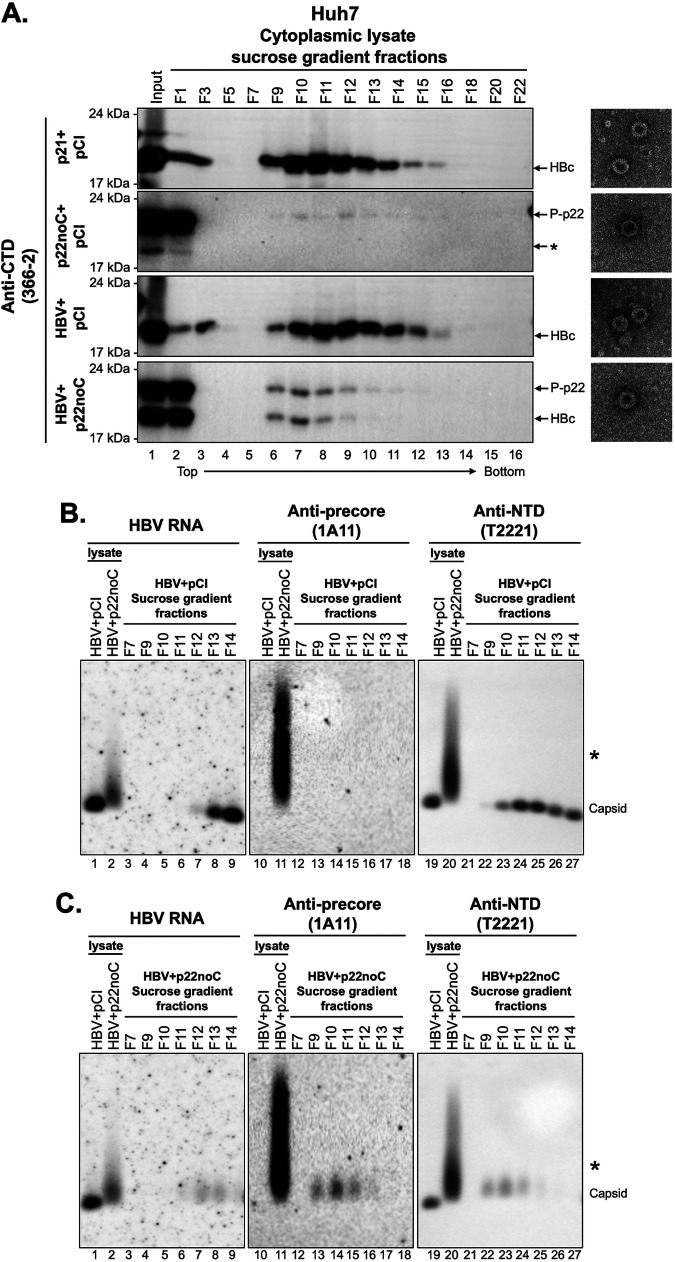
Analysis of intracellular capsids from transfected human hepatoma Huh7 cells. Huh7 cells were cotransfected with pCI-HBc (p21) or pCI-p22-noC and empty vector (pCI-empty) or the HBV replicon pCI-HBV-HBc and pCI-empty or pCI-p22-noC, as indicated, at a 1:1 mass ratio and harvested at 72 h posttransfection. Cytoplasmic lysates from transfected cells were fractionated by sucrose gradient ultracentrifugation, and fractions were collected from top to bottom. (A) The fractions were resolved by SDS-PAGE, followed by immunoblotting using the anti-precore/core CTD MAb 366-2 (left). Representative images of negatively stained capsids under transmission electron microscopy from each sample are shown on the right. Asterisks (*) indicate the degraded product of p22noC that was detected by Western blotting. (B and C) Cytoplasmic lysates from Huh7 cells cotransfected with pCI-HBV-HBc and pCI-empty (i.e., HBV+pCI) or pCI-HBV-HBc plus pCI-p22-noC (i.e., HBV+p22noC) were loaded together with the indicated fractions (7 to 14) from the sucrose gradient of HBV+pCI (B) or HBV+p22noC (C), resolved by NAGE, and transferred to a nitrocellulose membrane. HBV RNA was detected using the anti-sense HBV RNA probe followed by Western blotting of precore protein using anti-precore MAb 1A11 and precore/core protein using MAb T2221 on the same membrane sequentially. Asterisks (*) indicate the p22-RNA complex in the cytoplasmic lysate of HBV+p22noC-transfected cells.

As mentioned above, we detected weak HBV RNA signals comigrating with strong p22 protein signals above the capsids on NAGE when p22 was expressed in cells in the absence of HBc (Fig. 4A and E; Fig. S3A and E), suggesting that p22 might form complexes with RNA. After RNase A treatment, the weak RNA signal was lost and the p22 protein signal was evidently decreased in the absence of HBc expression (Fig. 4E and Fig. S3E, lane 11 versus 2), suggesting that p22 indeed formed RNA-protein complexes that migrated slower than capsids on NAGE and could be disrupted by RNase digestion. However, the HBV RNA signals from cells cotransfected with p22 or p25 plus the HBV replicon (i.e., in the presence of HBc) were not affected by RNase A treatment (Fig. 4E and Fig. S3E, top, lanes 13 to 18 versus lanes 4 to 9), just like the RNA signal detected when the replicon was transfected in the absence of p22 or p25 (Fig. 4E and Fig. S3E, top, lane 10 versus lane 1), suggesting that these RNAs were protected because they were packaged inside capsids containing p21. However, the p22-specific signal, as detected by the precore-specific monoclonal antibody (MAb) 1A11, decreased after RNase A treatment (Fig. 4E and Fig. S3E, middle, lanes 13 to 15 versus lanes 4 to 6), again indicating the disruption of the p22-RNA complex, which comigrated with the chimeric capsids and failed to protect the small amounts of HBV RNA (and possibly non-HBV RNA as well, which would not be detected by our assay). The loss of p22-specific signal following RNase digestion was less pronounced when the ratio of p22 protein to HBc was decreased (Fig. 4E and Fig. S3E, middle, compare lanes 15 and 6 versus lanes 13 and 4). Moreover, in the presence of p22, chimeric capsids migrated slower, and migration was slowed more when p22 was at higher levels than p21 (Fig. 4E and Fig. S3E, lanes 4 to 6 and lanes 13 to 15). The signals detected by the NTD MAb (T2221), representing both p22 and HBc, were not significantly affected by RNase digestion (Fig. 4E and Fig. S3E, bottom). From these results, it can be surmised that provided the chimeric capsids consisted of at least 50% p21, the RNA was protected from RNase digestion (see also Fig. 5).

### Analysis of chimeric capsids formed by cytosolic p22 and HBc.

To further demonstrate the formation of hybrid capsids by p22 and HBc, we performed sucrose gradient fractionation to separate capsid particles based on their size (Fig. 5). HBc that forms native capsids sedimented into the middle of the gradient (F9-16), with a small amount staying on top of the gradient (fractions 1 to 3) presumably as dimers (Fig. 5A, first and third panels). However, the majority of p22 localized at the top of the gradient (Fig. 5A, second panel), which is consistent with its inability to assemble efficiently into capsids. Interestingly, trace amounts of p22 sedimented into the capsid fractions, suggesting that p22 alone formed capsids, albeit much less efficiently than HBc. Like p22 expressed alone, when p22 and HBc were coexpressed, the majority of p22 remained at the top of the gradient, and, moreover, most HBc also stayed at the top of the gradient (Fig. 5A, fourth panel), an indication that p22 suppressed capsid formation by HBc. Using negative staining transmission electron microscopy (TEM), we were able to visualize capsids or capsid-like particles formed by p22 alone (Fig. 5A, second panel, right). These p22 capsids were of a similar size and shape as authentic capsids formed by HBc (Fig. 5A, right). Furthermore, using MAb 3120, which recognizes a structural epitope on the HBV capsid but not on the dimer (38), we were able to immunoprecipitate p22 from cells expressing p22 alone (Fig. S5, lane 2), further indicating that p22 forms capsid-like structures. In addition, in HepG2 cells cotransfected with p22 and p21 constructs, the amount of p22 immunoprecipitated using MAb 3120 was higher than p22 expressed alone, indicating the incorporation of p22 into p22-p21 chimeric capsids (Fig. S5, lane 5). This observation is in agreement with the sucrose gradient and TEM observations (Fig. 5). However, the unphosphorylated p22 derived from p25 was not precipitated by MAb 3120 (Fig. S5, lanes 3 and 6), suggesting that this species of p22, which is translocated to the ER lumen (28), does not assemble into capsids.

10.1128/mbio.03501-22.5 FIG S5 Immunoprecipitation of the precore protein p22 from transfected cells. HepG2 cells cultured in 10-cm dishes were cotransfected with pCI-HBc (p21), pCI-p22-noC, pCI-p25-noC, and pCI-empty (pCI) or pCI-HBV-HBc, as indicated, at a 1:1 mass ratio. Cytoplasmic lysates were collected at 72 h posttransfection and subjected to immunoprecipitation using the mouse anti-HBc MAb 3120 or the nonspecific mouse IgG (i.e., the isotype-matched negative control for immunoprecipitation). Inputs (unprecipitated) and precipitates were loaded equally and resolved by SDS-PAGE, followed by immunoblotting using rabbit anti-HBV precore/core CTD MAb 366-2. Download FIG S5, JPG file, 0.2 MB. Copyright © 2023 Hong et al. 2023 Hong et al. https://creativecommons.org/licenses/by/4.0/ This content is distributed under the terms of the Creative Commons Attribution 4.0 International license.

We detected capsid-associated pgRNA signals in fractions of sucrose gradients from cells cotransfected with p22 and the HBV replicon. Similar to the chimeric capsids resolved by NAGE without gradient fractionation (Fig. 4; Fig. S3), these RNA signals migrated slightly slower than capsids formed by HBc (Fig. 5B and C, lanes 2 and 6 to 9 versus lane 1). Furthermore, we detected the precore-specific signals in the fractions containing capsid-like particles with packaged pgRNA (Fig. 5B and C, lanes 16 to 18), further supporting that capsids containing p22 could package pgRNA. It was notable that whereas the RNA-containing capsids formed by HBc or HBc plus p22 sedimented to the same fractions (Fig. 5B and C, F12 to F14), empty capsids formed by HBc plus p22, which were the majority in both cases, sedimented slightly slower (Fig. 5C, F9 to F11) than those formed by HBc alone (Fig. 5B, F10 to F12). Thus, chimeric capsids formed by HBc plus p22 might be slightly smaller or lighter, but this requires further characterization.

### Precore proteins are part of HBV virions.

**FIG 6:**
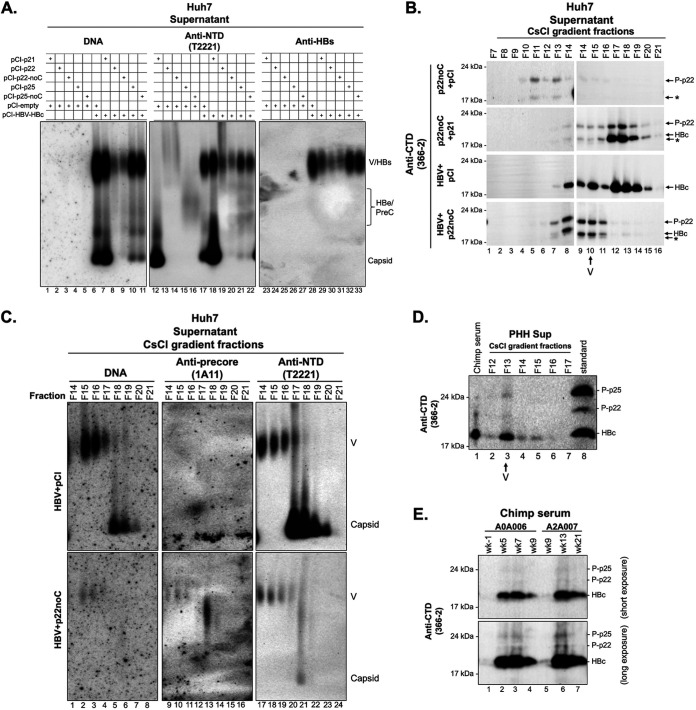
Analysis of HBV virions from transfected human hepatoma Huh7 cells, HBV-infected PHHs, and HBV-infected chimpanzees. (A) Huh7 cells cultured in 10-cm dishes were cotransfected with pCI-HBc (p21), pCI-p22, pCI-p22-noC, pCI-p25, pCI-p25-noC, and empty vector (pCI-empty) or the HBV replicon pCI-HBV-HBc, as indicated, at a 1:1 mass ratio. Cell culture supernatant was collected at 72 h posttransfection, concentrated (by 50×) by PEG precipitation, resolved by NAGE, and transferred to nitrocellulose membranes. HBV DNA and capsid (in both naked capsids and in virions) and envelope proteins were detected sequentially using a ^32^P-labeled HBV DNA probe, the anti-HBV precore/core NTD MAb T2221, and the anti-HBs polyclonal antibody (Virostat), respectively, on the same membrane. (B) Cell culture supernatant of transfected Huh7 cells was fractionated by CsCl gradient ultracentrifugation. Indicated fractions (fractions 7 to 21) were resolved by SDS-PAGE followed by immunoblotting with an anti-precore/core CTD antibody 366-2 for detecting p22 and HBc proteins. Fractions 7 to 14 and 14 to 21 were loaded onto two separate gels and processed in parallel. Fraction 14 was loaded on both gels as a reference to facilitate comparison of signals from the two gels. Fraction 15 was the peak of HBV DNA-containing virions as shown in C. (C) Fractions (14 to 21) from CsCl gradient ultracentrifugation were resolved by NAGE. HBV DNA, precore protein in virions, and precore/core proteins in virions were detected by a ^32^P-labeled HBV DNA probe, anti-precore MAb 1A11, and anti-precore/core NTD MAb T2221 sequentially on the same membrane. (D) Cell culture supernatants of genotype D HBV-infected PHHs collected and harvested at 10 dpi were fractionated by CsCl density gradient centrifugation. Fractions were resolved by high-resolution SDS-PAGE, along with the sera from the genotype D HBV-infected chimpanzee (number 1616, week 22) and *in vitro* translated p21, p22, and p25 proteins in the rabbit reticulocyte lysate. The precore and core proteins were detected by anti-precore/core CTD MAb 366-2. This image was a long-time exposure of Fig. 10B from our previous study (21). (E) Immunoblotting of HBV precore/core protein in genotype D HBV-infected chimpanzee sera by SDS-PAGE using anti-precore/core CTD-specific MAb 366-2. V, virion; HBe/PreC, secreted HBeAg and PreC protein.

As p22 was able to form chimeric capsids, some of which could package pgRNA and synthesize DNA, we were interested in determining the effects of intracellular precore proteins on virion secretion. We resolved the concentrated cell culture supernatant by NAGE and detected virion-associated DNA by Southern blotting, followed by Western blotting of virion-associated core protein. We found that cotransfection of p22 and HBV replicon reduced secretion of DNA-containing virions (Fig. 6A, lanes 8 and 9) and empty virions (Fig. 6A, lanes 19 and 20) in Huh7 cells, which could be accounted for by decreased intracellular capsid and encapsidated pgRNA levels (Fig. 4 and 5). Interestingly, we found that DNA-containing virions and empty virions were also slightly decreased when we cotransfected p25 and the HBV replicon (Fig. 6A). Additionally, release of naked capsids, whether empty or DNA containing, was reduced by p22 or p25 cotransfection more than that of virions, suggesting that precore expression could inhibit naked capsid release. In agreement with a recent study (26), expression of cytosolic p22 evidently decreased HBsAg secretion (Fig. 6A, lanes 30 and 31 versus lane 28), while expression of p25 only slightly decreased HBsAg secretion (Fig. 6A, lanes 32 and 33 versus lane 28). Interestingly, overexpression of HBc (p21) also decreased levels of HBsAg secretion (Fig. 6A, lane 29 versus lane 28). Our results suggest that the precore protein (most likely the cytosolic precore protein derivatives) inhibits HBV virion secretion, which is consistent with the results above showing that G1896A (i.e., precore-negative) HBV-infected cells showed increased DNA-containing virion secretion (Fig. 3).

The assembly of DNA-containing and empty chimeric capsids by p22 together with HBc raised the intriguing possibility that precore-containing chimeric capsids might be secreted in virions. To test this possibility, we fractionated cell culture supernatant from cells cotransfected with p22 and HBc or the HBV replicon by CsCl density gradient ultracentrifugation. When p22 was expressed alone, we could detect low levels of p22 in the fractions with lower density than virions (Fig. 6B, first panel), possibly in some form of vesicles like exosomes (36). However, when coexpressed with HBc, p22 sedimented into the high-density fractions containing naked capsids, consistent with the formation of intracellular chimeric capsids that were released into the culture supernatant of transfected cells (Fig. 6B, second panel), like authentic capsids formed by HBc alone (Fig. 6B, third panel). When p22 was expressed together with the HBV replicon, there was a dramatic decrease of naked capsid release (Fig. 6B, fourth panel), as already evident from NAGE analysis of the culture supernatant without CsCl gradient fractionation (Fig. 6A, lanes 8 to 9 and 19 to 20), confirming that p22 could suppress the release of naked capsids when envelope proteins were present. Furthermore, both p22 and HBc cosedimented into the virion fractions (Fig. 6B, lanes 7 to 10), suggesting that p22 was secreted in virions as part of the chimeric capsid consisting of both HBc and p22. Also, we resolved selected CsCl gradient fractions derived from the HBV replicon alone or the replicon plus p22 by NAGE. We found that regardless of p22 expression, the DNA-containing virion peak was in fraction 15, whereas the empty virion peak was in fraction 14 (Fig. 6C). Furthermore, the precore-specific protein (i.e., p22) comigrated on NAGE with virion-associated DNA and capsid signals from the p22 and HBV cotransfected group (Fig. 6C, bottom, middle panel). These results thus indicate that p22 is secreted in both empty and DNA-containing virions.

Given that the above results were obtained from HBV-transfected hepatoma cells overexpressing p22, we were interested in determining whether the precore proteins are also secreted in virions in the supernatant of HBV-infected PHHs and in the sera of HBV-infected chimpanzees. The CTD-specific MAb 366-2 detects HBc or precore proteins with a CTD but not the soluble HBeAg and PreC from the genotype D HBV used here, which lack the 366-2 epitope (18, 21). Thus, MAb 366-2 allows the differentiation of p22, which, as we have shown, is found in chimeric capsids and is part of secreted virions (Fig. 5 and 6A to C), from PreC, which is a secreted soluble protein. Using PHH supernatant fractionated on a CsCl density gradient (21), we could detect small amounts of p22 and p25 cosedimented with HBc to the peak virion fraction (Fig. 6D, lane 3, F13), indicating that the precore proteins p22 and p25 were indeed part of HBV virions secreted from HBV-infected PHHs. Furthermore, we also detected p22 and p25 in the sera of both HBV-infected chimpanzees (Fig. 6E), suggesting that these two cytosolic precore proteins could be incorporated into virions in HBV-infected chimpanzees *in vivo*.

## DISCUSSION

**FIG 7:**
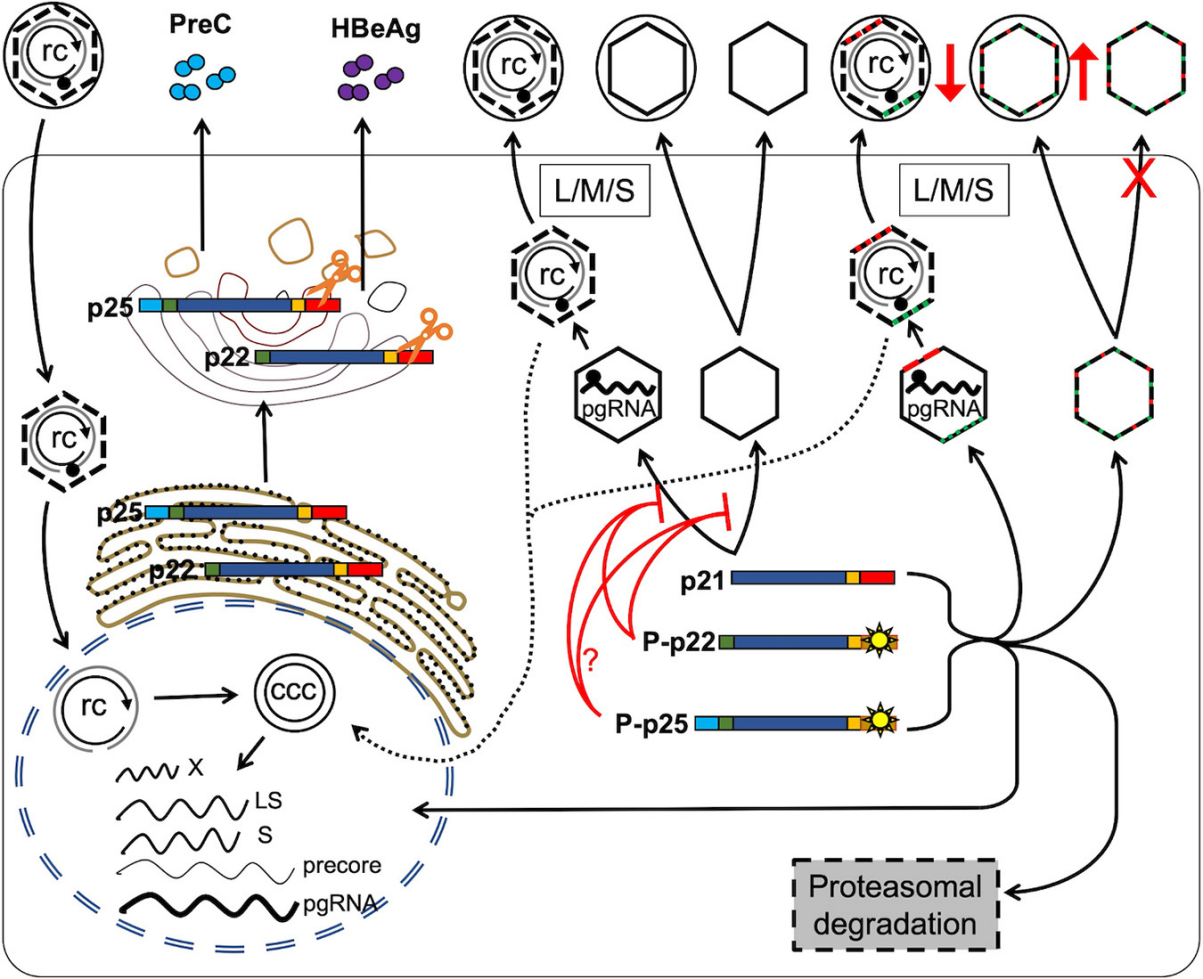
Model of HBV precore protein expression and functions in the HBV life cycle. The HBV life cycle is depicted briefly with the major replication steps outlined. During translation of the precore mRNA, the N-terminal signal peptide of the precore precursor p25 directs p25 to translocate into the ER lumen cotranslationally, with the signal peptide being cleaved off by the ER-associated signal peptidase, resulting in the production of p22 in the ER lumen. p22 traffics through the ER-Golgi secretory pathway, where the CTD of p22 is processed by the preprotein convertase (e.g., furin) to generate the secreted HBeAg. A small portion of p25 is translocated into the ER lumen without the removal of the N-terminal signal peptide and traffics similarly through the ER-Golgi secretory pathway, where its CTD is similarly processed before secretion as PreC. However, the abortive translocation of p25 after signal peptide cleavage or retrotranslocation of p22 results in cytosolic localization of p22 and its phosphorylation (denoted by the yellow suns). In addition, some p25 is never translocated to the ER and is also phosphorylated in the cytosol. A small amount of cytosolic P-p22 and P-p25, denoted by short red and green lines on the capsid symbols (hexagons), respectively, form chimeric capsids (empty and genome containing) with HBc and are secreted in virions. Also, cytosolic P-p22 and possibly P-p25, by forming heterocomplexes with HBc, inhibit capsid formation by HBc (denoted by long red lines), decrease HBc levels partially through the proteasomal degradation pathway, and stimulate nuclear localization of HBc. In addition, P-p22 and presumably P-p25 suppress (denoted by the downward arrow and cross) rcDNA-containing virion secretion and naked capsid release but increase (denoted by the upward arrow) empty virion secretion in HBV-infected hepatocytes.

We have demonstrated the presence of four intracellular precore-derived proteins, including phosphorylated and nonphosphorylated p22 and p25, in HBV-infected PHHs (Fig. 7). Although the precore protein is not essential for HBV replication (39), we found that precore protein derivatives caused decreases in HBc, which consequently resulted in a decrease in pgRNA packaging and DNA replication. Furthermore, the precore protein, specifically, the cytosolic precore protein derivatives, reduced complete HBV virion and naked capsid secretion but could stimulate empty virion secretion. We found that cytosolic precore proteins, p22 and p25, formed chimeric capsids with HBc and were furthermore secreted in virions by HBV DNA-transfected hepatoma cells, HBV-infected PHHs, and infected chimpanzees (Fig. 7). To our knowledge, this study is the first to characterize intracellular precore proteins and study their roles in HBV-infected PHHs and HBV-infected chimpanzees *in vivo*.

Our results revealed that unphosphorylated p22 and p25 are the processing intermediates of HBeAg and PreC, respectively, in HBV-infected PHHs and precore-transfected hepatoma cells (Fig. 2; Fig. S1). Aborted translocation or retrotranslocation of precore results in the presence of precore proteins in the cytosol (16, 23, 30). In agreement with a recent study (28), P-p22 and P-p25 were localized in the cytosol (Fig. 2F), and they were likely phosphorylated by a cytosolic protein kinase(s), whereas the precore processing intermediates of the secreted HBeAg and PreC (i.e., p22 and p25) were unphosphorylated due to their localization in the ER-Golgi secretory pathway. The detection of P-p25 in the cytoplasm of HBV-infected PHHs as well as precore-transfected hepatoma cells (Fig. 2; Fig. S1) indicates that the efficiencies of signal peptide recognition and ER translocation were also suboptimal for the precore precursor in authentic HBV-infected cells, possibly due to the cysteine residues within the signal peptide (24). Interestingly, a portion of phosphorylated precore proteins, P-p25 and P-p22, were also associated with membrane compartments in the absence of other HBV proteins (Fig. 2F), possibly with autophagosomes, as shown recently (36). The presence of a small amount of p22 in the supernatant of overexpressing cells as a low-density entity (Fig. 6B) may be the result of secretion of membrane-associated p22 via exosomes/vesicles. However, our previous studies failed to detect the low-density population of p22 in the sera of HBV-infected patients (21), potentially because much less cytosolic p22 was produced in HBV-infected human hepatocytes than in the p22-overexpressing cells. Interestingly, we were able to detect a low-density population of secreted HBeAg and PreC protein in the sera of chronically HBV-infected patients and woodchuck hepatitis virus (WHV)-infected woodchucks (21, 22). The nature of these secreted, low-density precore proteins and their potential functions warrant further study. Although similar precore derivatives were detected previously in precore-overexpressing hepatoma cells (24, 28, 29), our study is the first to detect intracellular precore proteins in HBV-infected PHHs, the most physiologically relevant cell culture system mimicking HBV infection in humans.

We found that PHHs infected with virions carrying the G1896A mutation, which does not express precore protein, showed a marked increase of intracellular HBc compared to PHHs infected with WT HBV (Fig. 3; Fig. S2). Previous studies of the HBV G1896A mutation showed increased pgRNA packaging and DNA replication in replicon-transfected cells (33, 34). A subsequent study using tandem HBV dimer-transfected cells also found a similar effect of the G1896A mutation, but in those transfection experiments, the effect appeared to be mainly due to the *cis* effect of the mutation on HBc translation from pgRNA and possibly the precore mRNA (40). However, we did not observe any major *cis* effect of the G1896A mutation on HBc expression from cells transfected with a replicon construct that supports pgRNA but not precore mRNA expression, suggesting that the increased HBc expression we observed in the G1896A-mutant-infected cells was not due to the influence of the mutation on HBc translation from pgRNA. However, we showed that the cytosolic p22 could form chimeric capsids with HBc but only at very low efficiency compared with HBc alone, and most HBc remained unassembled when coexpressed with cytosolic p22 (Fig. 5), indicating that cytosolic p22 inhibited capsid formation by HBc. Thus, although a small amount of p22 coassembled with HBc into capsids, our results suggest that most HBc was likely degraded either as p22-HBc heterocomplexes before assembly into chimeric capsids or as unstable chimeric capsids. This degradation was at least in part via proteasomes, as the decrease in HBc was alleviated in the presence of the proteasomal inhibitor MG132 (Fig. 4; Fig. S3 and S4). Also, in the presence of p22, some unassembled HBc and p22-HBc heterocomplexes were transported to the nucleus (Fig. S4). Therefore, the decrease in pgRNA packaging and DNA replication caused by the precore protein, as observed here (Fig. 4; Fig. S3) and previously (33, 34), is a consequence of reduced HBc expression and capsid assembly. However, it remains possible that the short distance between the stop codon introduced by the G1896A mutation and the downstream initiation codon of the core gene makes efficient translational termination and reinitiation on the mutant precore mRNA to synthesize HBc in G1896A HBV-infected hepatocytes possible (40). Thus, HBc translation from the G1896A-mutant precore mRNA could also contribute to increased HBc levels in mutant-infected cells. Reciprocally, loss of HBc reduced the levels of intracellular p25 and p22 in both HBV-infected PHHs and precore-transfected hepatoma cells (Fig. 2; Fig. S1). This is possible, in part, due to HBc interactions with these precore proteins, leading to their assembly into capsids or capsid-like complexes and hence their stabilization, but additional mechanisms may also be involved.

Here, we detected a small amount of capsid-like particles formed by cytosolic p22 alone that appeared similar to capsids formed by HBc under negative stain TEM (Fig. 5). Previous studies did not detect any capsids formed by the precore protein alone in human cells (21, 25), likely due to the lower sensitivity of detection in those studies. Artificial HBeAg expressed in Escherichia coli can form capsids under reducing conditions (41). In addition, the presence of precore-derived p22 and p25 in the cytosol and in virions secreted from HBV-infected cells (Fig. 6) suggests that cytosolic p25 might behave similar to cytosolic p22 in terms of its interactions with HBc and that p25 may also interact with p22.

Our results from HBV-infected PHHs and transfected human hepatoma cells suggest that precore proteins also inhibit the secretion of DNA-containing virions and release of naked capsids (Fig. 3, 6, and 7; Fig. S1). As cytosolic p22 and p25 can be incorporated into capsids (Fig. 5 and 6), these precore proteins might directly affect capsid interaction with the viral envelope proteins during virion morphogenesis. Whereas precore proteins inhibited complete virion secretion in both HBV-infected PHHs and HBV-infected HepG2-huNTCP cells, they only increased empty virion secretion in PHHs but showed no effects on empty virion secretion in HepG2-huNTCP cells (Fig. 3; Fig. S1 and S2). As HBsAg expression levels in HBV-infected HepG2-huNTCP cells are much lower than those in HBV-infected PHHs (42, 43), the different ratios of HBsAg to empty capsids in the different cells might thus be responsible for the differential effects of precore on empty virion secretion. However, the detailed mechanisms of differential empty virion secretion in precore-positive (WT) and precore-negative (G1896A) HBV-infected PHHs versus hepatoma cells remain to be elucidated. The reduction in naked capsid release caused by precore could be explained by the reduced levels of intracellular capsids as well as their enhanced secretion in virions. In contrast to HBV replicon-transfected hepatoma cells that are well known to secrete large quantities of naked capsids (6), HBV-infected HepG2-huNTCP cells released few such naked capsids (Fig. 3; Fig. S2), possibly because HBc expression in the replicon-transfected hepatoma cells is much higher than that from cccDNA in HBV-infected HepG2-huNTCP cells.

Interestingly, expression of cytosolic p22 decreased the secretion of HBsAg in transfected cells (Fig. 6), similar to a previous study showing that overexpression of precore (p25 and p22) from an HBV subgenotype A1 strain was associated with decreased HBsAg secretion in transfected cells (26). However, loss of precore expression in PHHs infected with the G1896A mutant did not affect HBsAg secretion (Fig. 3; Fig. S1), suggesting that any effect of precore on HBsAg secretion during natural infection is likely minimal due to low expression of the precore proteins. However, the HBV subgenotype A1 strain possesses a G1862T mutation in the precore region, which results in a valine-to-phenylalanine substitution that interferes with signal peptide cleavage and leads to impaired HBeAg secretion (44, 45). It is possible that accumulation of cytosolic p25 in this HBV subgenotype A1 strain could potentially explain its inhibitory effect on HBsAg secretion (44), perhaps even in infected hepatocytes *in vivo*, similar to the effect of overexpressed cytosolic p22 on HBsAg secretion. As shown by a previous study, the expression of precore proteins, including p25 and p22, could also decrease intracellular HBsAg levels (26), suggesting that precore may affect HBsAg expression or stability.

A previous study suggested that PreC (previously known as p22cr) is part of DNA-free HBV virions (20). However, as we recently reported, PreC shares the same size and density profiles as HBeAg, which are distinct from virions, and are not part of virions (21). Here, we found that PreC was secreted via the classical ER-Golgi secretory pathway, like HBeAg. HBeAg and PreC, located in the secretory pathway, are unable to assemble into capsids due the oxidizing environment (41, 46) nor coassemble with HBc, which is located in the cytosol. Therefore, neither PreC nor HBeAg are likely to be secreted in virions. Instead, our results demonstrate that the cytosolic precore proteins p22 and possibly p25 could be coassembled with HBc into replication-competent nucleocapsids and empty capsids intracellularly and could be secreted in DNA-containing and empty virions, respectively (Fig. 5 and 6), which renders HBV virions (both DNA containing and empty) secreted from WT (precore-positive) HBV-infected hepatocytes highly heterogeneous (Fig. 7). Therefore, future studies are warranted to determine the roles of the virion-associated precore proteins, for example, in viral entry and infection processes.

In addition to directly affecting the HBV life cycle, as we found in the current study, the cytosolic precore-derived p22 is thought to inhibit host innate immune responses to facilitate HBV persistence (25, 47). Virion-associated p22 or p25 might also play a role in modulating the host innate immune response. Moreover, the secreted HBeAg is also thought to have immunoregulatory functions to facilitate viral persistence (10, 11). In particular, the maternal HBeAg is thought to suppress HBV-specific T cell activities, resulting in HBV persistence during vertical (mother to child) transmission (48, 49). HBV evolved from Nackednaviruses, non-enveloped DNA viruses related to hepadnaviruses, with the envelope proteins acquired later (50). Remarkably, the precore protein emerged even later than the envelope proteins (14, 50). Here, our results suggest that the precore protein can function as a negative regulator of naked capsid secretion, a function that may have been selected positively during HBV evolution. The HBV capsid is highly immunogenic and can stimulate a robust antibody response, and the secretion of HBV naked capsids may cause antibody-mediated cell lysis, which may be associated with fulminant hepatitis (51). Similarly, high levels of HBV DNA replication may be cytopathic for the infected hepatocytes, as shown for the duck hepatitis B virus (52), or trigger strong host immune responses. The downregulation of HBc expression and consequent reduction of HBV replication by the precore protein may also favor viral persistence. Future studies will be required to further elucidate the functions of the various precore proteins (intracellular or extracellular, soluble or particulate) in viral replication and pathogenesis.

## MATERIALS AND METHODS

### Plasmids.

The wild-type (WT) HBV replication-competent plasmid replicon, pCIΔA-HBV-HBc-WT (genotype D), was described previously (53). pCIΔA-HBV-HBc-C(-), the HBV replicon construct defective in HBc expression, was constructed by substitution of the core initiation codon (ATG) with GTG. pCIΔA-HBV-HBc-C(-), when complemented by cotransfecting with pCI-HBc to provide HBc in *trans*, supports HBV replication. pCIΔA-HBV-HBc-G1896A was constructed by substitution of the −28 codon (TGG) with the TAG stop codon in the precore-specific region by PCR-directed mutagenesis. The HBV precore/core protein expression constructs pCI-HBc, pCI-p22, and pCI-p25 have been described previously (21). The precore expression constructs pCI-p22-noC and pCI-p25-noC were constructed by subcloning the p25-noC (full-length precore) and p22-noC (with a deletion of the signal peptide from −2 to −19 amino acids of p25) coding sequences from pCIΔA-HBV-HBc-C(-) in the pCI vector (Promega), respectively.

### Cell cultures.

Human hepatoma cell lines HepG2, HepG2-huNTCP, and Huh7 were cultured in Dulbecco’s modified Eagle’s medium-F12 (DMEM-F12) supplemented with 10% fetal bovine serum (FBS) (HyClone) and 50 μg/mL penicillin-streptomycin (15, 21). PHHs were purchased from BioreclamationIVT (Westbury, NY). Cryopreserved PHHs were thawed and seeded in cell plating medium (Life Technologies, CA) at 0.25 million cells per well in 24-well collagen I-coated plates (Corning, NY) and were maintained in maintenance medium containing 2% dimethyl sulfoxide (DMSO) and 2% FBS, as previously described (21, 54).

### Transient transfection.

Transient transfection of HepG2 and Huh7 cells was done as previously described (15, 54). HepG2 or Huh7 cells seeded in 35-mm, 60-mm, or 100-mm dishes were transfected with 2 μg (total), 4 μg (total), or 12 μg (total) of plasmid using X-tremeGENE HP DNA transfection reagent (Roche) or FuGENE6 (Promega), respectively. When performing two-plasmid cotransfection, a mass ratio of 1:1 of each plasmid was used (21, 54). All transfection experiments were repeated between two and four times.

### HBV infection.

HBV infection in HepG2-huNTCP and PHH cells was carried out as previously described (53, 54). The cells were infected with HBV inoculum prepared from transfected HepG2 cells at a multiplicity of infection (MOI) of ca. 400 in the presence of 2% DMSO and 4% polyethylene glycol 8000 (PEG 8000). After 16 h, the viral inoculum was removed, and the cells were washed three times with warm phosphate-buffered saline (PBS) before adding fresh medium containing 2% DMSO. Eight days postinfection, the cells were harvested and subjected to protein or DNA analysis.

### Western blotting.

Cell lysates were resolved by 12.5% sodium dodecyl sulfate-polyacrylamide gel electrophoresis (SDS-PAGE). The mouse monoclonal antibody (MAb) clone 1A11 (a gift from Peter Revill, Peter Doherty Institute for Infection and Immunity) was used for specific detection of precore-related proteins (Fig. 1). The mouse MAb T2221 (2AHC24, Tokyo Future Style, Japan) that is specific for the precore/core NTD and the rabbit MAb 366-2 that is specific for the precore/core CTD were used for the detection of precore and core proteins (Fig. 1) (21). The rabbit polyclonal antibody against HBsAg (1811, Virostat, Portland, ME, USA) was used for the detection of HBV surface proteins. Horseradish peroxidase-conjugated anti-mouse IgG (31439, Invitrogen, USA) or anti-rabbit IgG (4010-05, Southern Biotech, USA) diluted at 1:5,000 or 1:10,000, respectively, were used as the secondary antibodies.

### Analysis of viral DNA from infected cells or transfected cells.

The nucleocapsid-associated HBV DNA (i.e., core DNA) was released from the cytoplasmic lysate containing viral nucleocapsids by treatment with 0.5% SDS and 0.6 mg/mL Protease K (Invitrogen) at 37°C for 1 h (53). Hirt extraction was used for purifying HBV protein-free DNAs (PF-DNAs) (15, 53, 54). PF-DNA was subsequently digested with exonucleases I and III (ExoI and ExoIII) as previously described (55). Viral DNAs were resolved on 1.2% agarose gels and detected by a [^32^P]dGTP-labeled DNA probe targeting the entire length of the HBV genome.

### Native agarose gel electrophoresis (NAGE) assay for analyzing assembled capsid and RNA packaging.

To analyze the levels of assembled capsids and the amount of pgRNA packaged inside nucleocapsids, cytoplasmic lysate was resolved on a 1% native agarose gel. Following the transfer of viral particles from the gel to a nitrocellulose membrane, a [^32^P]UTP-labeled HBV antisense RNA riboprobe was used to detect encapsidated HBV RNAs. Subsequently, capsids were detected by the mouse anti-HBc MAb (T2221) on the same membrane (15, 54).

### Assay for virion secretion.

Cell culture supernatant was precipitated with PEG 8000 and concentrated by 50 times or concentrated by 25 times using 10-kDa cutoff ultrafiltration units, as previously described (21, 56). The concentrated supernatant was subjected to DNase I digestion to remove plasmid DNAs and resolved on a 1% native agarose gel (15). Following the transfer of viral particles from the gel to a nitrocellulose membrane, a [^32^P]dGTP-labeled HBV DNA probe was used to detect HBV DNA in the viral particles (15).

### Analysis of HBV capsids by sucrose gradient ultracentrifugation.

The cytoplasmic lysates of transfected cells were fractionated by sucrose gradient ultracentrifugation as previously described (21, 22), with minor modifications. One milliliter of the lysate was first treated with 2.3 mM magnesium acetate (MgOAc) and 400 μg/mL DNase I at 37°C for 30 min to remove the plasmid DNA, followed by treatment with 20 mM EDTA and 200 μg/mL RNase A at 37°C for 60 min. After removal of the precipitates by a brief centrifugation, the supernatant was layered on top of a 15 to 30% linear sucrose gradient containing 1× HCB2 buffer (20 mM Tris [pH 7.5], 50 mM NaCl, 1 mM EDTA, 0.01% [vol/vol] Triton X-100, and 0.1% NP-40) in a 12-mL ultracentrifugation tube and centrifuged at 40,000 rpm for 2 h and 45 min at 4°C in a Beckman SW41Ti rotor. Fractions (500 μL each) were collected from the top to bottom.

### Analysis of HBV virion secretion by CsCl density gradient centrifugation.

Virions and subviral particles in concentrated cell culture supernatants were purified by CsCl density gradient ultracentrifugation as previously described (21, 22). Fractions (200 μL each) were analyzed by native agarose gel electrophoresis for virion particles and by SDS-PAGE for analysis of viral proteins.

### Negative stain TEM.

Samples from fractions 9 to 12 after sucrose gradient ultracentrifugation were concentrated 20-fold by using the 10-kDa cutoff ultrafiltration unit. Samples (4 μL) were then applied to a glow-discharged copper grid coated with continuous carbon film and stained with 2% (wt/vol) uranyl acetate. Images were acquired using a JEM-2100 equipped with Gatan UltraScan 4000, as previously described (57).
