# Supplementary figures and images for: Characterization of Intracellular Precore-Derived Proteins and Their Functions in Hepatitis B Virus-Infected Human Hepatocytes
Source: mBio. 2023 Jan 30;14(1):e03501-22. doi: 10.1128/mbio.03501-22 (PMC9973328; doi:10.1128/mbio.03501-22)

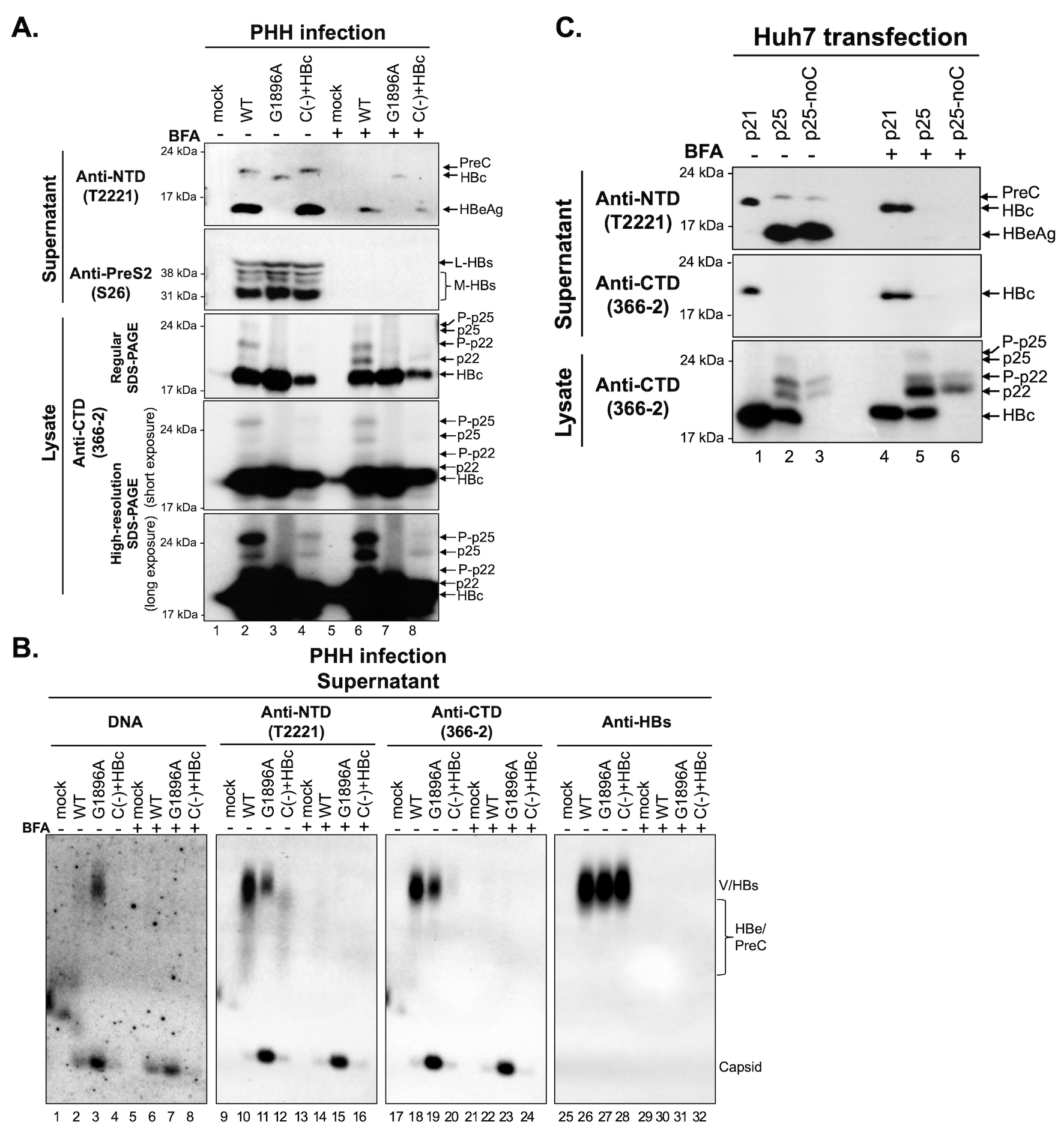

Supplement: FIG S1 [file mbio.03501-22-s0001.jpg]

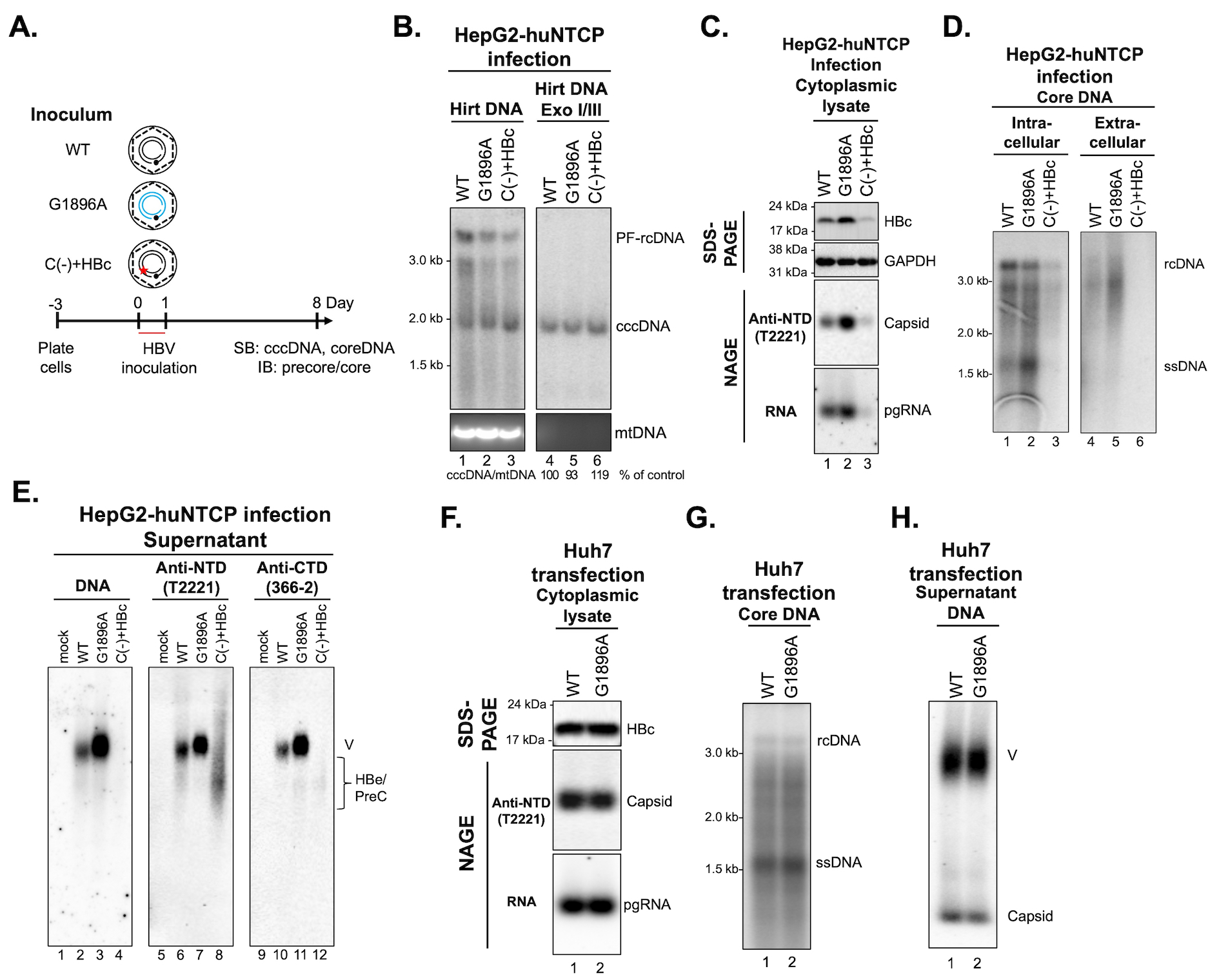

Supplement: FIG S2 [file mbio.03501-22-s0002.jpg]

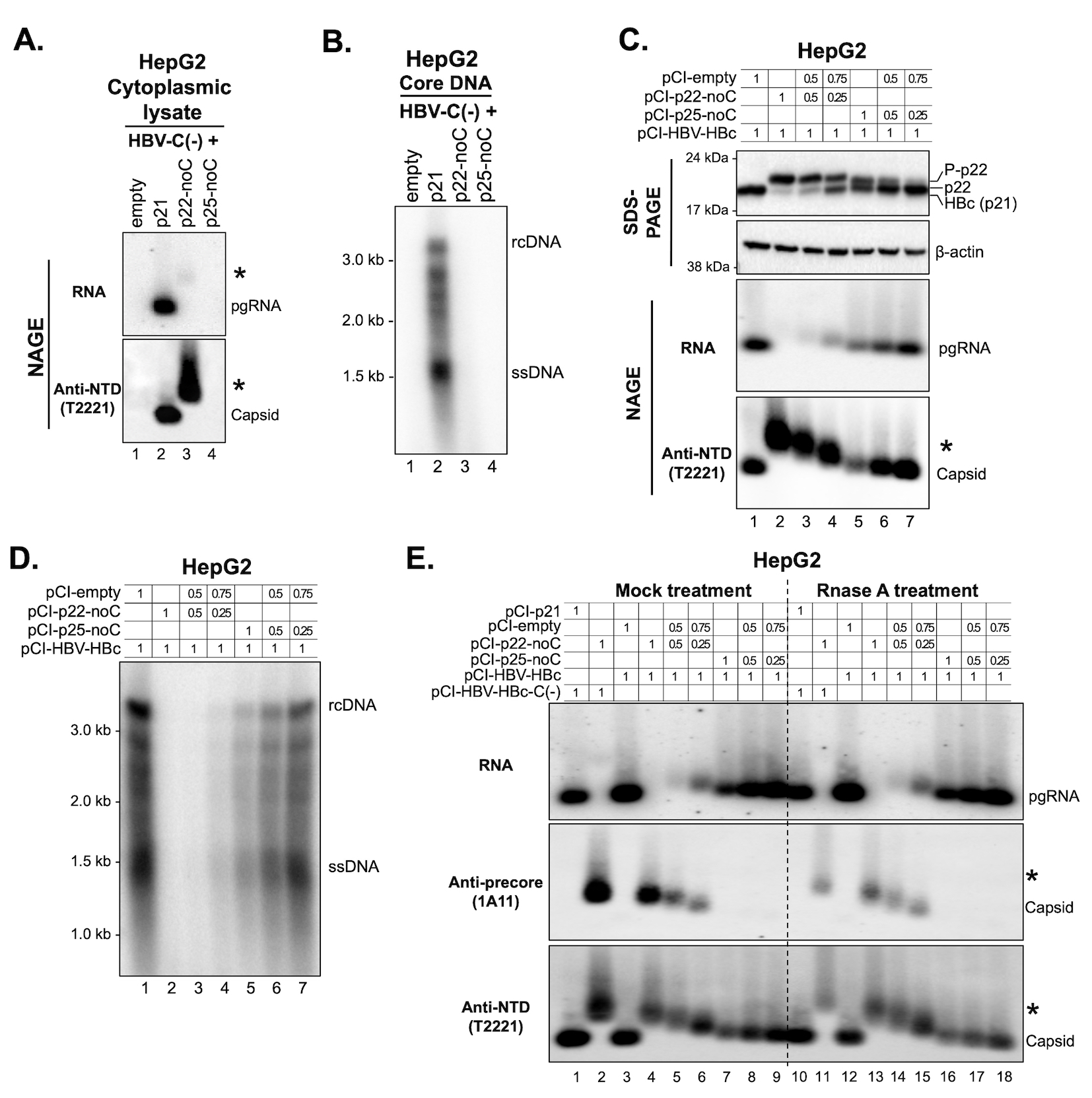

Supplement: FIG S3 [file mbio.03501-22-s0003.jpg]

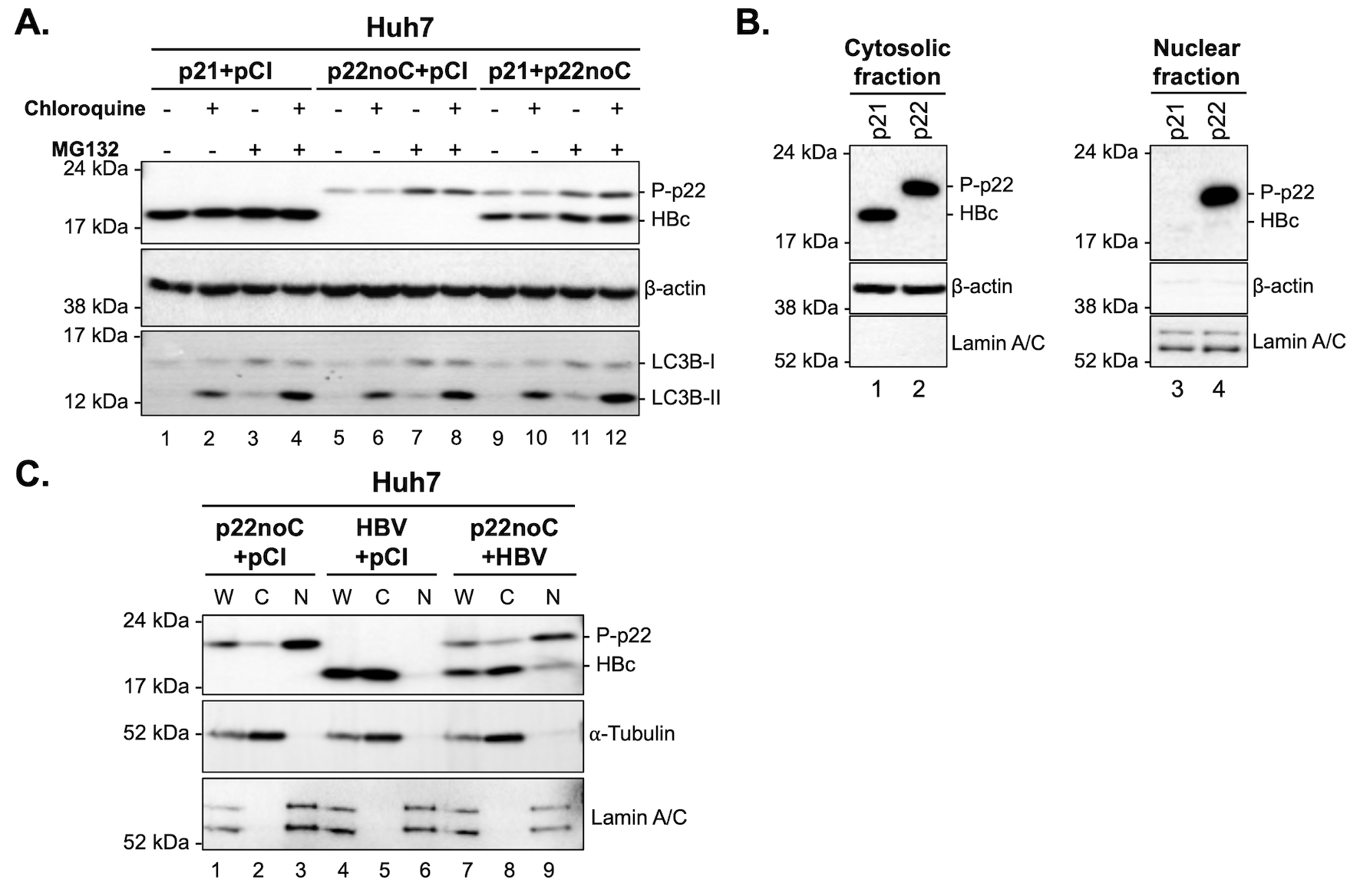

Supplement: FIG S4 [file mbio.03501-22-s0004.jpg]

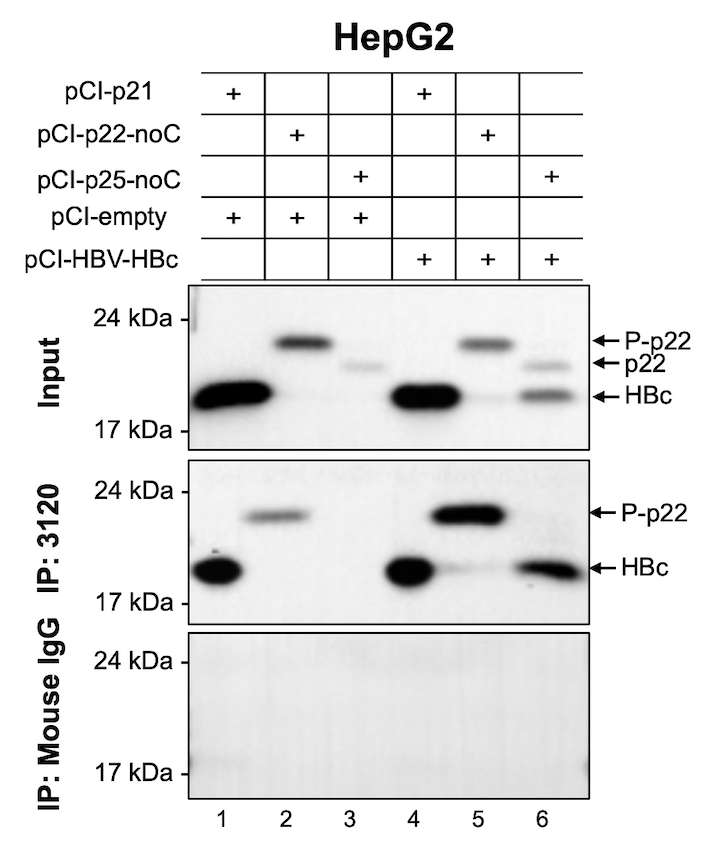

Supplement: FIG S5 [file mbio.03501-22-s0005.jpg]
